# Influence of Treated Surface Proportion on the Antibacterial Performance of UV-Activated Hydroxyapatite–Magnesium Phosphate–Zinc Oxide Coating on Magnesium Alloys

**DOI:** 10.3390/jfb17030133

**Published:** 2026-03-09

**Authors:** Purificación Tamurejo-Alonso, Juan Manuel Casares-López, Federico Rafael García-Galván, Juan Antonio Constantino, Amparo M. Gallardo-Moreno, Juan Carlos Galván, Miguel Ángel Pacha-Olivenza, M. Luisa González-Martín

**Affiliations:** 1 Center for Biomedical Research Networking: Bioengineering, Biomaterials and Nanomedicine (CIBER-BBN), 06006 Badajoz, Spain; ptamurejo@unex.es (P.T.-A.); juconstantic@unex.es (J.A.C.); amparogm@unex.es (A.M.G.-M.); mlglez@unex.es (M.L.G.-M.); 2 Department of Applied Physics, Faculty of Sciences, University of Extremadura, 06006 Badajoz, Spain; jmcasares@unex.es; 3 University Campus of Badajoz, University Institute for Biosanitary Research of Extremadura (INUBE), 06006 Badajoz, Spain; 4 Department of Biomedical Sciences, Faculty of Medicine and Health Sciences, University of Extremadura, 06006 Badajoz, Spain; 5 Department of Mechanical Engineering, Chemical Engineering and Industrial Design, Polytechnic University of Madrid (UPM), 28012 Madrid, Spain; fr.garcia.galvan@upm.es; 6 Department of Surface Engineering, Corrosion and Durability, National Center for Metallurgical Research (CENIM-CSIC), 28040 Madrid, Spain; jcgalvan@cenim.csic.es; 7 Department of Medical-Surgical Therapeutics, Faculty of Medicine and Health Sciences, University of Extremadura, 06006 Badajoz, Spain; 8 Orthopaedic Surgery and Traumatology Department, University Hospital Complex of Badajoz, 06006 Badajoz, Spain

**Keywords:** magnesium alloys, electrodeposition, partial surface coverage, ultraviolet treatment, infection, antimicrobial, surface damage, zinc oxide

## Abstract

Surface damage occurring during surgery can compromise coating integrity, leaving exposed areas susceptible to bacterial colonization. However, the impact of partial coating loss on antibacterial performance has not yet been investigated. In this work, a multifunctional UV-activated coating composed of hydroxyapatite, magnesium phosphate, and zinc oxide (HMZ) was developed and electrodeposited onto AZ31 and MgCa magnesium alloys. Its antibacterial efficacy against *Staphylococcus aureus* and *Escherichia coli* was evaluated under three conditions: adhered bacteria, planktonic cells, and biofilm. In the absence of UV activation, coated surfaces exhibited no significant antibacterial activity. In contrast, fully coated and UV-activated surfaces achieved bacterial reductions above 98% in all scenarios. Surfaces with 60% coverage showed antibacterial efficacy equivalent to that of fully coated surfaces, even against established biofilm. Surfaces with 30% coverage also exhibited moderate activity, particularly against adhered and planktonic bacteria. These results demonstrate that full surface coverage is not required to preserve the coating’s antibacterial effectiveness. This strategy provides a clinically relevant solution to maintain antibacterial protection even when coating integrity is compromised.

## 1. Introduction

Population ageing and modern lifestyle demands have increased the need for surgical interventions to treat bone injuries [1]. These interventions require biomaterials that are not only biocompatible [2] but also offer the necessary mechanical resistance for adequate structural support [3]. Traditionally, permanent materials such as titanium, cobalt-chrome, and steel alloys have been employed. However, their mechanical properties do not align well with those of natural bone, which could compromise adjacent tissue, increase the risk of secondary fractures [4], and increase the need for second surgeries [5,6,7].

An alternative is biodegradable metals, which enhance biocompatibility and more closely mimic the mechanical characteristics of bone, thereby avoiding the complications associated with permanent implants. Biodegradable metals such as magnesium and its alloys are particularly promising for orthopedic applications [8,9,10]. Specifically, the alloys of the AZ series (Mg-Al-Zn), and in particular AZ31 (Mg-3Al-1Zn), are the most studied both in vitro and in vivo [11,12]. Magnesium is an essential trace element, and the Mg^2+^ ions released during degradation are naturally utilized by the body without causing toxicity or adverse effects [13,14,15,16]. Recent in vivo studies using Mg-Zn-Ca alloys have confirmed their resorbable nature and antibacterial performance in bone defect models [17]. Moreover, high local concentrations of Mg^2+^ have shown cytotoxic effects on osteosarcoma cells, inhibiting proliferation and migration [18]. F. Witte et al. [19] conducted corrosion tests on various magnesium alloys implanted in guinea pig femurs. Their results suggest that the corrosion products were either phagocytosed by multinucleated cells or dissolved and excreted by the kidneys without inducing toxicity.

Despite their interesting properties, magnesium alloys also present certain drawbacks. One of the main concerns is their relatively high degradation rate in physiological environments [20,21,22], which is strongly influenced by the pH and the presence of chloride ions [23]. This degradation rate, often higher in vitro than in vivo [24,25], may prematurely reduce the mechanical integrity of the alloy and cause the release of hydrogen in quantities that exceed the body’s absorption capacity [24,25,26,27,28]. These effects could hinder bone regeneration [29] and negatively affect blood flow [30].

Although the safety of magnesium-based implants has been demonstrated in various in vivo studies [19,31,32,33,34,35,36,37,38], localized corrosion phenomena such as pitting may still occur. These events can initiate crack formation in the material and accelerate degradation, leading to a significant loss of structural integrity [6,7,11].

Among the different approaches to partially overcome this drawback, those focusing on surface modification stand out [39,40,41,42,43,44]. Specifically, the electrodeposition technique consists of the generation of a metallic coating on a base material, thanks to the electrochemical reduction of the electroactive species dissolved in an electrolyte [45]. In this regard, calcium phosphate coating in the form of hydroxyapatite (HA) has aroused great interest due to its similarity to bone apatite. Its porous structure has been shown to promote bone growth [46]. More recent studies comparing inorganic surface treatments on Mg1Ca alloys found that crystalline hydroxyapatite coatings provided superior corrosion resistance and lower cytotoxicity compared to phosphate or aluminum oxide coatings [47]. Also, mixed coatings, such as combinations of hydroxyapatite, magnesium and zinc phosphate, have also shown potential to enhance corrosion resistance and boost osseointegration. In this context, Huang et al. [48] obtained excellent corrosion results and good biocompatibility with mouse fibroblasts with this coating.

It is widely known that one of the most serious complications in the use of implants is infections [49,50,51]. Bacterial colonization of biomedical devices begins with the physical adhesion of bacteria to the material’s surface. This phenomenon, initially reversible, becomes irreversible as biochemical interactions between the microorganisms and the material culminate in biofilm formation. These multicellular structures enhance the bacteria’s resistance to antibiotics, acting as a virulence mechanism that significantly complicates the treatment of implant-associated infections [52].

In this context, magnesium- and zinc-based coatings have gained increasing attention. Several studies have shown that the degradation products of magnesium-based materials can impair bacterial viability and adhesion, thus contributing to infection prevention [53,54,55,56]. This effect has been partially attributed to the local pH increase caused by magnesium ion release [55,57,58], which disrupts bacterial electrochemical gradients and reduces adenosine triphosphate (ATP) synthesis through excessive H^+^ consumption [59].

In addition to pH elevation, some authors propose that high concentrations of Mg^2+^ ions generate osmotic stress, further compromising bacterial survival [53,57,60,61,62]. Similarly, Zn^2+^ ion release during coating degradation exerts a bacteriostatic effect [63]. The negatively charged peptidoglycan layer of bacterial cell walls binds these cations, reducing bacterial metabolic activity without inducing cell death.

An additional feature of zinc-based coatings, and in particular zinc oxide, is their response to ultraviolet (UV) radiation. Zinc oxide is an n-type semiconductor with a bandgap energy of 3.37 eV [64], comparable to titanium dioxide at 3.2 eV [65,66], which falls within the UV-C radiation range. When activated, ZnO generates reactive oxygen species (ROS), with biocidal activity linked to the production of H_2_O_2_. This compound can penetrate cytoplasmic membranes [45,67,68,69,70,71,72,73,74,75], leading to lipid peroxidation. Bakhshesdhi-Rad et al. [76] demonstrated antibacterial activity by incorporating TiO_2_ and zinc-doped hydroxyapatite in a magnesium alloy. Xie et al. [76] reported that ZnO nanostructures exhibited a rapid bactericidal effect through photocatalysis under UV irradiation, achieving bacterial reductions exceeding 97% for *E. coli* and 94.9% for *S. aureus* within one minute. Furthermore, this effect of ZnO can persist for hours after the UV source is removed, enhancing its antibacterial properties [77,78].

During surgical implantation, defects may occur in the surface coatings. These damages include material loss, cracking, or delamination, especially in high-friction areas such as threads [79,80]. Such alterations compromise coating integrity and expose the underlying metallic substrate to the physiological environment [19,81]. In magnesium alloys, the exposed areas are highly reactive, which can accelerate local degradation and promote bacterial adhesion [12,82]. These defects are particularly relevant in coatings designed with antibacterial functionality, as partial loss may compromise implant biocompatibility [83,84] and reduce their effectiveness against microbial colonization.

This research evaluates, for the first time, the antibacterial efficacy of a UV-activated surface treatment after partial coating loss, as may occur during the surgical insertion of biodegradable magnesium implants. In the present work, a two-step coating process combining electrodeposition and a subsequent alkaline treatment was employed to obtain a hybrid HMZ coating (hydroxyapatite–magnesium phosphate–zinc oxide). This approach allowed the preparation of stable, crystalline coatings suitable for evaluating efficacy under partial coverage and UV activation conditions. The coating was designed to combine osteoconductive and antibacterial properties, the latter being activated by UV irradiation. To address this, surfaces with controlled coverage levels of 30%, 60%, and 100% were fabricated and tested. Experiments were carried out on two magnesium alloys, AZ31B and Mg0.6Ca, against two clinically relevant bacterial strains: *S. aureus* (Gram-positive) and *E. coli* (Gram-negative). Antibacterial activity was assessed on both planktonic and sessile bacteria, as well as on biofilm formation. This experimental strategy simulates a realistic surgical scenario and allows the minimum coverage threshold needed to preserve the antimicrobial performance of this functional coating to be determined.

## 2. Materials and Methods

### 2.1. Substrate Preparation

Two magnesium alloys, AZ31B (AZ31) and Mg0.6Ca (MgCa), were used in the form of 25 mm diameter and 2 mm thick discs. Specimens were mechanically polished consecutively with 3 µm and 1 µm diamond paste, rinsed with ethanol, and dried with hot air. Due to magnesium’s high affinity for the ambient atmosphere, the maximum exposure time to air was limited to 1 h before specimen characterization.

### 2.2. Surface Preparation

HMZ coatings were electrodeposited on AZ31 discs following a procedure previously described for AZ31 [85], and on MgCa using an adapted protocol. A two-electrode cell with a 110 mm gap was operated at 37 ± 1 °C. The magnesium alloy acted as the working electrode, while a platinum electrode served as the counter electrode.

For full surface treatment (AZ31-100 and MgCa-100), current densities of 5 A/cm^2^ and 15 A/cm^2^ were applied, with electrodeposition times of 7200 s and 3600 s, respectively.

For partial surface treatments (AZ31-30, AZ31-60, MgCa-30, and MgCa-60), specific regions were masked to prevent coating, exposing only 30% or 60% of the surface to the electrolyte. These samples were designed with a striped pattern composed of alternating 3 mm-wide coated and uncoated bands. The masked areas were defined using a high-performance insulating tape resistant to strong electrolytes and elevated temperature. This material is inert under the electrodeposition conditions employed and does not leach impurities or modify local current distribution. XPS analysis performed on samples previously covered with the tape confirmed the absence of surface contamination or foreign elements). The coating–uncoated interface obtained by this masking strategy exhibited well-defined boundaries and homogeneous thickness. In these cases, the current density and electrodeposition time were maintained constant, while the total applied current was adjusted according to the exposed surface area.

The electrolyte solution consisted of 0.042 mol/L Ca(NO_3_)_2_·4H_2_O, 0.042 mol/L Mg(NO_3_)_2_·6H_2_O, 0.042 mol/L Zn(NO_3_)_2_·6H_2_O, 0.078 mol/L (NH_4_)H_2_PO_4_, 0.100 mol/L NaNO_3_, and 8.820 mol/L H_2_O_2_. The pH was adjusted to 4 using H_3_PO_4_.

After electrodeposition, an alkaline heat treatment was applied to transform the coatings into dense and crystalline layers. This treatment involved immersing the electrodeposited surfaces in a 0.1 mol/L NaOH solution at 80 °C for 4 h. During electrodeposition, local alkalisation promotes Zn(OH)_2_ and phosphate precursor formation rather than direct ZnO. The subsequent alkaline treatment promotes Zn(OH)_2_ dehydration into ZnO and stabilizes calcium phosphate as crystalline hydroxyapatite. Finally, the samples were rinsed with distilled water and dried at 50 °C for 1 h in a vacuum oven.

### 2.3. Exposure to Ultraviolet Light Source

A set of untreated samples, a set of partially treated samples, and another set of fully treated samples were exposed to a UV-C source for 24 h (referred to with the subscript UV). This exposure period was sufficient to ensure a post-radiation effect of the coatings capable of affecting bacterial viability [77,78]. A high-pressure mercury lamp (Philips, Ibérica, Spain) was used as the UV source, with a dominant emission line at 254 nm (UV-C range). A high-pressure mercury lamp (Philips, Ibérica, Spain) was used as the UV source, with a main emission peak of 254 nm (85–90%) and a secondary peak of 185 nm (5–10%), producing ozone that is filtered by the lamp glass. The irradiation period of 24 h at 254 nm and 4.2 mW cm^−2^ was chosen based on prior optimization studies in our laboratory and in the literature, ensuring complete ZnO photoactivation and stable ROS production across the entire surface. Shorter exposure times could activate the surface only partially, but the chosen protocol guarantees reproducibility and comparability among all tested conditions. The samples were positioned 100 mm from the light source, centred, and exposed to an intensity of approximately 4.2 mW/cm^2^, receiving a mean dose of 169.8 ± 3.7 J cm^−2^ measured by a cosine-corrected sensor connected to a dosimeter UV-MAT (OPSYTEC Dr. Gröbel GmbH-Germany, Ettlingen, Germany).

### 2.4. Physicochemical Characterization of Surfaces

#### 2.4.1. Chemical Composition of the Coating

The surface chemical composition of the samples was analyzed using X-ray Photoelectron Spectroscopy (XPS). XPS measurements were performed using a PHI 5000 VersaProbe II (PHI, Lafayette, LA, USA) equipped with a monochromatic AlKα X-ray source (1486.6 eV) with a spot size of 100 μm and 26.6 W power. High-resolution spectra were acquired for C1s, P2p, O1s, Mg1s, Ca2p, and Zn2p_3/2_ for each sample.

#### 2.4.2. Crystalline Structure of the Coating

The crystalline structure of the coatings was determined using X-Ray Diffraction (XRD) on a Bruker D8 Advance diffractometer (Bruker, Ettlingen, Germany) with Bragg–Brentano geometry and CuKα radiation (λ = 1.5406 Å). The angular range was set between 10° and 60°, with a step size of 0.02° and a scanning rate of 1° min^−1^. Peak interpretation was conducted using standards provided by the Joint Committee on Powder Diffraction Standards (JCPDS) [86], corresponding to hydroxyapatite (09-0432), magnesium phosphate (33-0876) and zinc oxide (89-1397).

#### 2.4.3. Morphology, Roughness, and Other Topographical Parameters of the Coating

A 3D optical profilometer (DCM8, Leica Microsystems, Barcelona, Spain) was used to evaluate surface roughness before and after coating. Topographical parameters were obtained using two external software programmes: one for acquisition (LeicaSCAN DCM8 v.6.6.9.1, Sensofar Tech. S.L., Terrassa, Spain) and one for analysis (LeicaMap Premium 8.1, Mountains Tech. Digital Surf, Spain). Surface texture images were analyzed according to roughness parameters provided by ISO 25178 [87] and EUR 15178N standards [88]. Surface morphology was further analyzed using a Scanning Electron Microscope (SEM, Quanta 200FEG, FEI, Eindhoven, Netherlands) with an accelerating voltage of 20 kV, a working distance of 9.5 mm, and 6500× magnification. The elemental composition of the coatings was subsequently analyzed by Energy Dispersive X-ray Spectroscopy (EDX), performed at 15 keV and integrated with the SEM system. EDS spectra were acquired in area-scan mode at 2000× magnification from representative surface regions of the coatings.

#### 2.4.4. Surface Electrical Properties

The electrical properties of the surfaces were determined through zeta potential (ξ) measurements. Zeta potential was obtained using streaming current measurements with an Electrokinetic Analyzer (SurPass 3, Anton Paar KG, Graz, Austria). An adjustable sample holder was used; the electrokinetic channel was formed with a standard material, polyvinylidene fluoride, and the samples under study. The electrolyte used was 1 mM KCl, prepared with MilliQ water of 18.2 MΩ·cm resistivity and a pH of 7.2. Zeta potential was calculated using the Helmholtz–Smoluchowski equation. Corrections associated with asymmetrical cells were undertaken [89].

#### 2.4.5. Release of Mg^2+^ and pH Change

The release of Mg^2+^ ions from all surfaces was quantified using inductively coupled plasma mass spectrometry (ICP-MS) with an Agilent 7900 ICP-MS system (Agilent Technologies, Santa Clara, CA, USA). The samples were in contact with phosphate-buffered saline (PBS, pH 6.8) for 30, 60, 120, 180, 300 min and 24 h, and with tryptic soy broth (TSB, pH 6.5, Becton Dickinson, Franklin Lakes, NJ, USA) for 24 h. The experimental setup was similar to that used in the bacterial assays (Section 2.6, Section 2.7 and Section 2.8) to allow for comparative results, but without the presence of bacteria. Additionally, pH measurements were recorded at the same time points using a LAQUAtwin-pH-33 metre (Horiba Scientific, Kyoto, Japan).

In addition, Zn^2+^ release was evaluated by ICP-MS under the same experimental conditions. In all cases, Zn^2+^ readings were below the instrument detection limit (<0.05 mg/L), including partial coverage and UV-irradiated surfaces.

#### 2.4.6. Corrosion Test

Electrochemical impedance spectroscopy (EIS) measurements were performed on the magnesium alloys during immersion tests in PBS solution at times of 2 h and 24 h to study corrosion behaviour. Measurements were performed using a potentiostat/galvanostat (PGSTAT4000, Metrohm Autolab, Utrech, The Netherlands) coupled with a frequency response analyser (FRA) and a three-electrode electrochemical cell [90]. A platinum mesh and an Ag/AgCl (3 M NaCl) electrode served as the counter and reference electrodes, respectively. The test specimen was connected as the working electrode, with an exposed surface area of 4.90 cm^2^. To minimize external interference, the electrochemical cell was housed within a Faraday cage. The impedance spectra were acquired by applying a logarithmic frequency sweep from 100 kHz to 10 mHz, recording ten data points per decade. A sinusoidal perturbation of ±10 mV was applied relative to the open-circuit potential (OCP). The OCP was monitored for 5 min before and after each EIS measurement to confirm its stability. The resulting impedance spectra were interpreted using equivalent electrical circuit models. Data fitting and analysis were performed with ZView software, version 4.0i (Scribner Associates, Inc., Southern Pines, NC, USA).

### 2.5. Bacterial Culture

The bacterial strains selected for the assays were *Staphylococcus aureus* ATCC 29213 (Gram-positive) and *Escherichia coli* ATCC 25922 (Gram-negative), both obtained from the American Type Culture Collection (ATCC). The strains were preserved at −80 °C using porous beads (Microbank, ProLab Diagnostics, Round Rock, TX, USA) until use. For maintenance, they were subcultured on blood agar plates (Oxoid, Ltd., Madrid, Spain) and subsequently incubated in TSB under agitation (100 rpm) at 37 °C for 14 h. This incubation time had been previously validated to ensure that the bacteria reached the late exponential growth phase. After incubation, the bacterial suspension was adjusted to a concentration of 10^8^ CFU/mL. For assays evaluating sessile and planktonic bacteria without further growth, the suspension was prepared in PBS. For biofilm formation tests, the bacteria were resuspended in fresh TSB.

### 2.6. Bacterial Adhesion and Antibacterial Activity on the Substrate

#### 2.6.1. Staining Technique

The Live/Dead staining method was applied to fully treated and non-treated surfaces, but not to the rest. This was done to identify significant damage to bacterial membrane integrity on fully coated surfaces, as a baseline for the analysis of partially coated surfaces. One millilitre of bacterial suspension was deposited on each surface and incubated at 37 °C under orbital shaking at 20 rpm (Heidolph Rotomax 120, Heidolph Electro GmbH, Schwabach, Germany) for 30, 60, 120, and 180 min. Following each incubation period, the samples were carefully retrieved and rinsed by sequential immersion in individual beakers containing fresh PBS to remove non-adherent bacteria. The adhered bacteria were then stained using the Live/Dead Baclight L-7012 kit (Invitrogen SA, Barcelona, Spain), following the manufacturer’s protocol. Viability and bacterial quantification were assessed by epifluorescence microscopy (Eclipse Ci, Nikon, Tokyo, Japan). Bacteria emitting red-orange fluorescence were classified as damaged, while those exhibiting green fluorescence were considered viable. Enumeration was performed using NIS-Elements BR 4.10 software (Nikon Instruments Inc., Melville, NY, USA). The percentage of damaged bacteria (%D) was calculated as follows:

(1) % D = ((T − V)/T) × 100

where T represents the total number of adhered bacteria, and V represents the number of viable bacteria.

#### 2.6.2. Serial Dilution Technique

The serial dilution technique was used to assess the viability of sessile microorganisms on surfaces. The incubation time for the 1 mL bacterial suspension was established based on the point at which 100% bacterial damage was observed on the fully treated surfaces after UV-C irradiation, as determined in the adhesion experiments described in Section 2.6.1. After the specified times, the substrates were removed and washed by immersion in separate beakers filled with fresh PBS to eliminate non-adherent microorganisms. The surfaces were then placed in contact with 3 mL of PBS and sonicated for 3 min to detach the bacteria adhered to the surface. The resulting bacterial suspension was centrifuged at 3000 rpm for 5 min, and the supernatant was removed. The pellet was washed twice with 3 mL of sterile PBS and resuspended in 1 mL of sterile PBS. This microbial extraction protocol was verified by inspecting all surfaces under microscopy to confirm the absence of any remaining adhered bacteria. The viability of the suspended bacteria from the pellet was assessed using the serial dilution method on agar plates. The antibacterial rate (%AR) was calculated using the following equation:

(2) % AR = ((N1 − N2)/N1) × 100

where N1 and N2 represent the cultured colony counts obtained from the initial bacterial suspension (N1) and from the suspension after remaining bacterial adhesion to the surface (N2).

### 2.7. Antibacterial Activity of Planktonic Bacteria

The antibacterial effect on non-adherent bacteria suspended near the studied surfaces was also analyzed. One millilitre of bacterial suspension was deposited on each surface. Samples were incubated at 37 °C with orbital shaking at 20 rpm. Incubation times corresponded to those at which 100% damage had been previously observed in sessile bacteria and were increased until 100% damage was reached in planktonic bacteria. These times were 120 and 180 min for *E. coli*, and 180 and 300 min for *S. aureus*. Bacterial viability in the suspension was determined using the serial dilution and plating method. The rate was calculated according to Equation (2), where N2 now refers to planktonic bacteria present in the suspension after contact with the surface.

### 2.8. Biofilm Formation

Biofilm formation on the analyzed surfaces was assessed through ATP quantification. For this purpose, 1 mL of bacterial suspension prepared in TSB was deposited on each surface and incubated for 24 h at 37 °C under orbital shaking at 20 rpm. After incubation, non-adherent bacteria were removed by gently rinsing the surfaces twice with sterile TSB. The number of viable bacteria within the biofilm was determined using the BacTiter-Glo microbial cell viability assay (Promega Corporation, Madison, WI, USA), according to the manufacturer’s guidelines. This technique estimates the metabolic activity of bacterial cells by measuring ATP. The BacTiter-Glo reagent was added directly onto each sample, allowing it to react in the dark for 15 min under gentle agitation (20 rpm). After the reaction, the supernatant was transferred to white, flat-bottom, 96-well polystyrene microplates (Greiner Bio-One, Frickenhausen, Germany), and luminescence was measured using a luminometer (Microplate Fluorescent Reader FLX 800, Bio-Tek Instruments, Winooski, VT, USA). The percentage of biofilm production relative to the uncoated AZ31 or MgCa substrates (%B) was calculated using the formula

(3) % B = ((B1 − B2)/B1) × 100

where B1 and B2 represent ATP production quantified as Relative Light Units (RLU) on the non-electrodeposited (B1) and electrodeposited (B2) samples.

### 2.9. Statistical Analysis

All experiments were conducted in triplicate, with technical duplicates included in microbiological assays. Descriptive statistics and subsequent data analysis were performed using R software 4.2.2 (R Core Team (2022). R: A language and environment for statistical computing. R Foundation for Statistical Computing, Vienna, Austria). Data are presented as mean ± standard deviation. Normality was assessed using the Shapiro–Wilk test (*n* < 50) and the Kolmogorov–Smirnov test (*n* ≥ 50). In the case of topographical and initial bacterial adhesion information, when data met the normality and homocedasticity (Barlett’s test) assumptions, two-way analysis of variance (ANOVA) was applied to compare group means between different alloys and bacterial strains, followed by Tukey’s Honest Significant Difference (HSD) post hoc test for pairwise comparisons. Statistical significance was set at *p* < 0.05.

## 3. Results

### 3.1. Surface Characterization

#### 3.1.1. XPS Analysis

**Figure 1:**
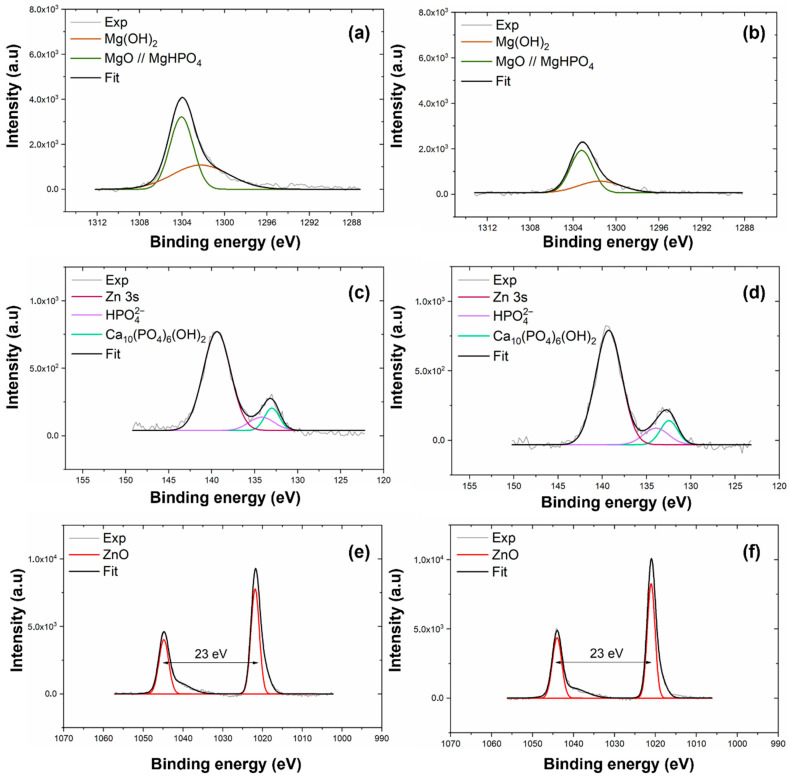
High-resolution XPS spectra of Mg1s, P2p, and Zn2p_3/2_ levels for coatings electrodeposited on AZ31 (**a**,**c**,**e**) and MgCa (**b**,**d**,**f**).

**Figure A1:**
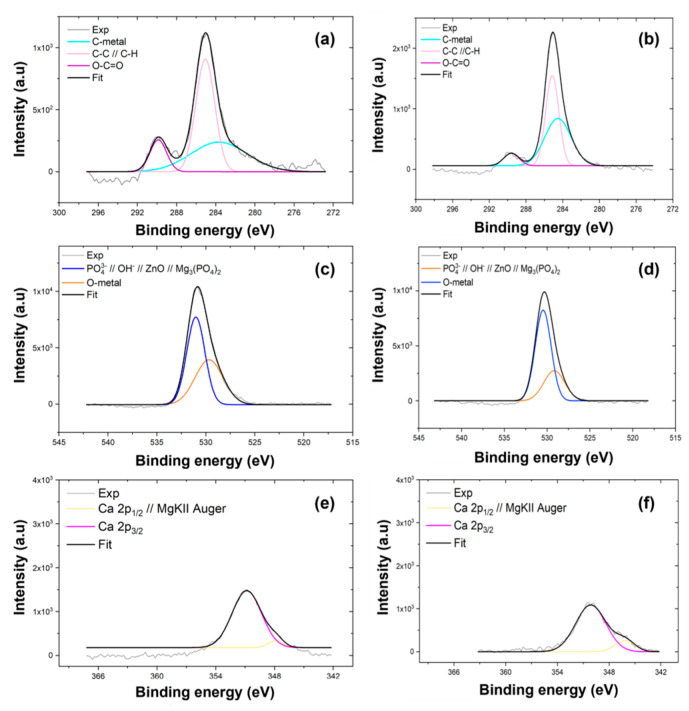
High-resolution XPS spectra of C1s, O1s, and Ca2p levels for coatings electrodeposited on AZ31-100 (a,c,e) and MgCa-100 (b,d,f).

XPS was employed to determine the chemical composition of the coating electrodeposited on AZ31 and MgCa alloys. High-resolution spectra were recorded for the C1s, O1s, Mg1s, P2p, Ca2p, and Zn2p_3/2_ levels. Figure 1 shows the Mg1s, P2p, and Zn2p spectra for the coatings deposited on AZ31 (a, c, e) and MgCa (b, d, f) samples. Details of the C1s, O1s, and Ca2p spectra deconvolution for these samples are provided in Appendix A (Figure A1).

The deconvolution of the Mg1s signal for both coatings, shown in Figure 1a,b, reveals two components. The first component, at 1302.3 eV, corresponds to MgO [91]. The second component, at 1304.0 eV, is attributed to Mg_3_(PO_4_)_2_ formation [92,93]. The interpretation of the Mg1s components was supported by the O1s spectra (c,d, Appendix A), which exhibited peaks at 530–531 eV and 532 eV, corresponding to O-metal/O-P and O-H/MgO bonds, respectively. These results confirm the coexistence of phosphate, oxide, and hydroxide species within the HMZ-coating.

High-resolution spectra in the energy range 140–125 eV, shown in Figure 1c,d, reveal two signals for both coatings. The first signal, centred at 133.3 eV, corresponds to the P2p level. The second signal, at 139.4 eV, is assigned to the Zn3s level, associated with ZnO [94]. Deconvolution of the P2p signal reveals two contributions. The first, at 133.0 eV for AZ31 and 132.5 eV for MgCa, is assigned to HA formation [95]. The second, at 134.2 eV for AZ31 and 133.9 eV for MgCa, is attributed to phosphate groups (${PO}_{4}^{3-}$) within the coating [94,96,97].

The Zn2p region, shown in Figure 1e,f, displays the Zn2p_3/2_ and Zn2p_1/2_ doublet with a spin–orbit separation of 23 eV. Deconvolution reveals two contributions for each coating. For AZ31, binding energies of 1021.9 eV and 1044.9 eV correspond to Zn atoms occupying regular ZnO lattice sites [98]. For MgCa, similar lattice positions are detected at 1021.1 eV and 1044.0 eV. A second contribution is identified at 1020.2 eV for both AZ31 and MgCa, and at 1041.9 eV for AZ31 and 1040.9 eV for MgCa. This second component is also associated with Zn–O bonds, although some authors attribute these shifts to oxygen vacancies or variations in zinc coordination within the crystalline structure [99,100].

#### 3.1.2. X-Ray Diffraction Analysis (XRD)

**Figure 2:**
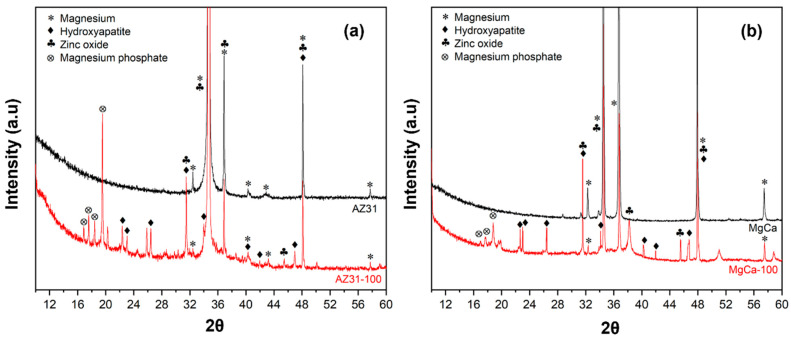
X-ray diffraction (XRD) patterns of coating on AZ31 (**a**) and MgCa (**b**) surfaces.

Different peaks from the XRD measurements from the electrodeposited coverages on AZ31 and on MgCa materials are shown in Figure 2. The diffraction peaks at 25.9°, 31.8°, and 32.2° correspond to hydroxyapatite (JCPDS card 09-0432). Reflections at 21.8° and 31.1° match magnesium phosphate (Mg_3_(PO_4_)_2_, (JCPDS card 33-0876). Peaks at 31.7°, 34.4°, and 36.2° confirm the presence of ZnO (JCPDS standard 89-1397) [86].

Some overlapping reflections were noted between hydroxyapatite and magnesium phosphate, which are common in multi-phase coatings. This overlap was particularly evident in the region between 31° and 32°, where peaks from both phases may contribute to the observed intensity.

#### 3.1.3. Morphology and Topography

**Figure 3:**
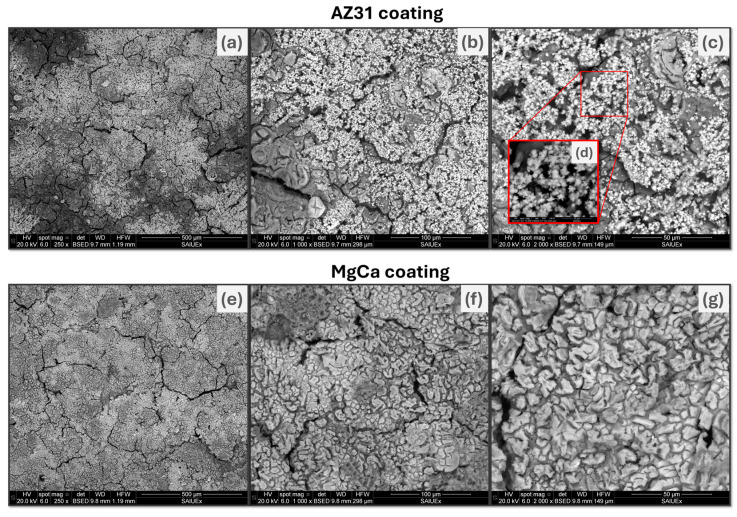
SEM images of coating on AZ31-100 (**top row**) and MgCa-100 (**bottom row**) at magnifications of 250× (**a**,**e**), 1000× (**b**,**f**), and 2000× (**c**,**g**). An inset (**d**) at 10,000× magnification highlights the flower-like morphology of the coating on AZ31-100, characteristic of ZnO.

Figure 3 shows SEM images of the surface morphology of the fully electrodeposited coating on AZ31 (AZ31-100) (a–d) and MgCa (MgCa-100) (e–g). At 250× magnification (a), the coating on AZ31-100 exhibits a broad distribution of crystals over a more compact base, with irregularly distributed cracks. This continuous layer may result from the unification of growth nuclei that overlap during electrodeposition. The spatial arrangement of crystals becomes clearer at 1000× (b) and 2000× (c) magnifications. At 10,000× magnification (d), the crystals exhibit a flower-like morphology, characteristic of ZnO [101,102,103].

In contrast, the coating on MgCa-100 exhibits a homogeneous surface texture at 250× magnification (e), with irregular cracks. At 1000× magnification (f), raised irregular structures are visible. At 2000× magnification (g), the surface appears rough, with compact, densely clustered structures. At 2000× magnification, platelet-like structures, typically associated with ZnO, are observed [104].

**Figure 4:**
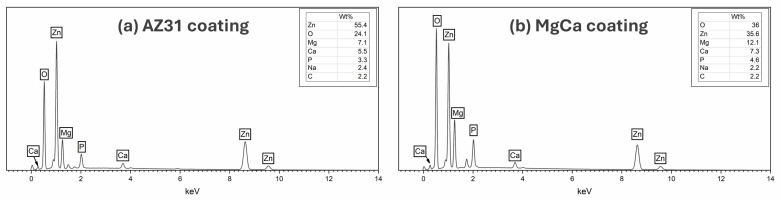
EDX spectra of coating on AZ31 (**a**) and MgCa (**b**) showing elemental composition. Spectra were acquired in area-scan mode at 2000× magnification from representative surface regions.

Figure 4 presents the EDX spectra for the coatings on AZ31-100 (a) and MgCa-100 (b). Both coatings contain Zn, O, Mg, Ca, P, Na, and C. The Ca/P ratios are 1.67 and 1.55 in AZ31-100 and MgCa-100, respectively, consistent with stoichiometric hydroxyapatite [105]. Although Ca/P ratios were calculated, they should be interpreted with caution for samples on the MgCa substrate, since part of the detected Ca may originate from the alloy itself.

#### 3.1.4. Surface Texture Analysis

The surface topography analysis of mirror-polished and HMZ coating on surfaces of AZ31 (AZ31-100) and MgCa (MgCa-100) is presented through relevant surface texture parameters, Root Mean Square Roughness (Sq), Maximum Peak Height (Sp), Maximum Valley Depth (Sv), Surface Skewness (Ssk), and Surface Kurtosis (Sku); the hybrid parameter Developed Interfacial Area Ratio (Sdr); and the spatial parameter Texture Aspect Ratio (Str). Table 1 includes these parameters at 10× magnification. Complete profilometry texture analysis is provided in Appendix A.

After polishing, AZ31 and MgCa surfaces appear smooth, with low Sq values, but with more and deeper valleys than peaks, as indicated by the negative skewness and higher Sv than Sp values. Also, a kurtosis parameter greater than 3 suggests that the topographical features of both surfaces are predominantly sharp. Nevertheless, the Developed Interfacial Area Ratio is low for both materials, around two percent. On the other hand, Str values below 0.3 indicate some anisotropy in both surfaces, probably related to scratching marks from the polishing process. It is worth noting that, despite the experimental uncertainty making it difficult to discern differences between the two materials, MgCa appears to be rougher than AZ31.

As expected, after electrodeposition induces a significant modification of the surface texture, reflected in the marked changes in all textural parameters. The results of the textural parameters reflect changes in surface topography that are similar for both alloys. Sq, Sp, and Sv increase considerably, but the height and depth of peaks and valleys, respectively, become much more similar between them than if they are compared in the polished condition, particularly for MgCa-100. Additionally, Ssk, Sku, and Str indicate that electrodeposition generates a topographically homogeneous surface, with any directionality from polishing effectively removed. As expected, Sdr increases considerably after electrodeposition, especially for AZ31-100.

#### 3.1.5. Zeta Potential

Zeta potential measurements were performed on polished and electrodeposited AZ31 and MgCa alloys. The uncoated AZ31 surface exhibited a zeta potential of 10 ± 5 mV, indicating a positive surface charge associated with the presence of metallic cations.

After electrodeposition, AZ31-100 showed a significant shift to a negative zeta potential of −24 ± 5 mV. This change of approximately −35 ± 10 mV is likely due to the incorporation of phosphate groups containing magnesium and calcium, which carry a negative charge under aqueous conditions.

The uncoated MgCa surface presented a zeta potential of 49 ± 19 mV, indicating a highly positive charge with substantial variability, suggesting heterogeneity at the surface level.

After coating, MgCa-100 displayed a reduced zeta potential of 29 ± 3 mV. Although the surface charge remained positive, the reduction of approximately −20 mV indicates the presence and effect of negatively charged groups incorporated during electrodeposition.

#### 3.1.6. pH Change and Release of Mg^2+^ Ions

**Figure 5:**
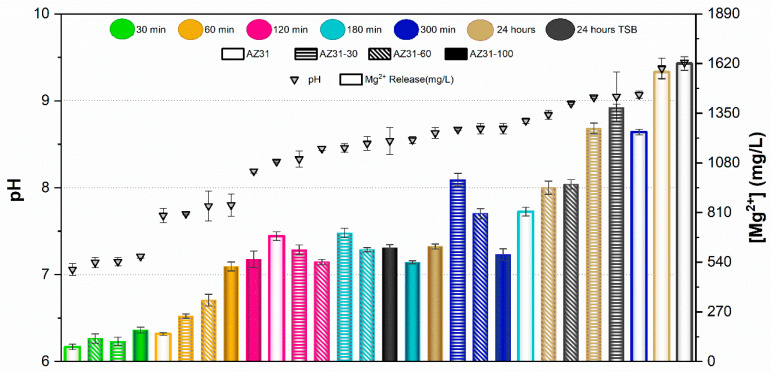
pH and Mg^2+^ concentration released from AZ31 with different proportions of treated surface at various contact times. Values are expressed as mean ± standard deviation of three independent technical replicates (*n* = 3).

**Figure 6:**
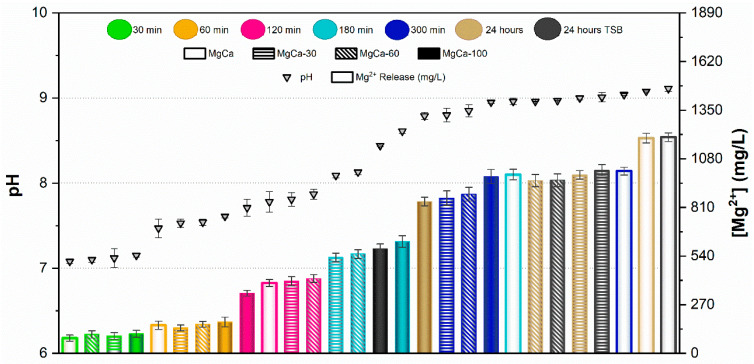
pH and Mg^2+^ concentration released from MgCa with different proportions of treated surface at various contact times. Values are expressed as mean ± standard deviation of three independent technical replicates (*n* = 3).

Results of the pH measurements and the concentration of Mg^2+^ ions released from samples after immersion for 30, 60, 120, 180, and 300 min in PBS are presented in Figure 5 and Figure 6 for AZ31 and MgCa, respectively. Results after 24 h immersion in TSB are also included in these figures for both materials.

In the case of AZ31, pH values remained below 8 for samples immersed up to 60 min in PBS. The concentration of Mg^2+^ ions released increased with the proportion of surface treatment. For immersion times of 120 and 180 min, pH ranged between 8 and 8.5. In this range, no significant differences were observed in ion release between different treatment percentages, except for the untreated surface at 180 min, which exhibited higher pH and ion release than the other samples. pH values above 8.5 were recorded after 300 min and 24 h of immersion in PBS. In these conditions, ion release decreased as the proportion of electrodeposited surface increased. In general, fully covered samples (full bars in Figure 5) showed the lowest Mg^2+^ concentrations in PBS, with values remaining nearly constant regardless of immersion time. Conversely, the uncovered alloy (empty bars in Figure 5) released the highest amount of Mg^2+^ ions at each time point, increasing with immersion time. Partially treated surfaces showed intermediate behaviour between uncovered and fully covered samples, according to their percentage of coverage. pH values and ion concentrations after 24 h immersion in TSB were similar to those observed in PBS for each coverage condition.

In the case of the MgCa alloy (Figure 6), pH evolution was smoother than for AZ31. pH remained below 8 for immersion periods up to 120 min, with similar ion release across all surface coverage percentages. Ion concentrations were comparable after 30 and 60 min and slightly higher after 120 min. For longer immersion times (300 min and 24 h), pH stabilized around 9 for all samples, regardless of surface coverage. For this alloy, partially treated surfaces yielded similar pH and ion release at each immersion time. As observed for AZ31, immersion in TSB and PBS produced comparable pH and ion release, except for the covered sample, which exhibited higher values in TSB than in PBS.

The ion release behaviour was primarily governed by the corrosion activity of the underlying substrate and the protective capacity of the HMZ coating. Uncoated surfaces exhibited rapid Mg^2+^ release due to direct electrolyte interaction with the alloy, while the electrodeposited coating acted as a physical and chemical barrier that limited ion diffusion. The partial coatings showed intermediate responses, consistent with exposed surface fraction. Differences between AZ31 and MgCa mainly reflect their intrinsic corrosion kinetics and alloy composition, with MgCa exhibiting smoother pH evolution owing to its higher calcium content and lower corrosion reactivity.

The comparison between UV-irradiated and non-irradiated surfaces showed similar pH values and Mg^2+^ concentrations under all conditions. These results confirm that UV exposure does not significantly alter Mg^2+^ release or the alkaline environment generated during coating degradation.

#### 3.1.7. Corrosion Analysis

**Figure 7:**
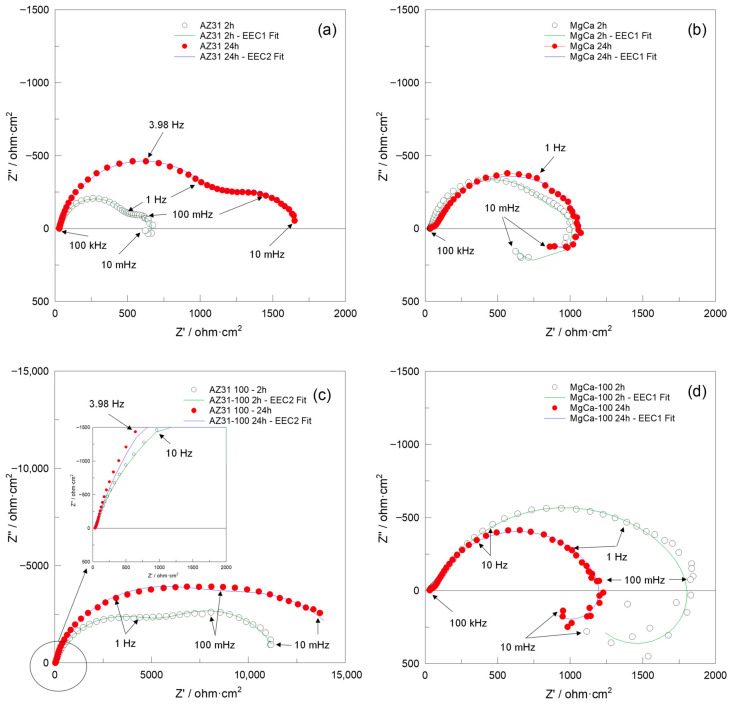
Nyquist plots recorded over the 100 kHz–10 mHz frequency range, along with fit results obtained using the EEC1 and EEC2 equivalent circuits shown in Figure 9, for (**a**) AZ31, (**b**) MgCa, (**c**) AZ31-100, and (**d**) MgCa-100 samples after 2 and 24 h of immersion in PBS solution.

**Figure 8:**
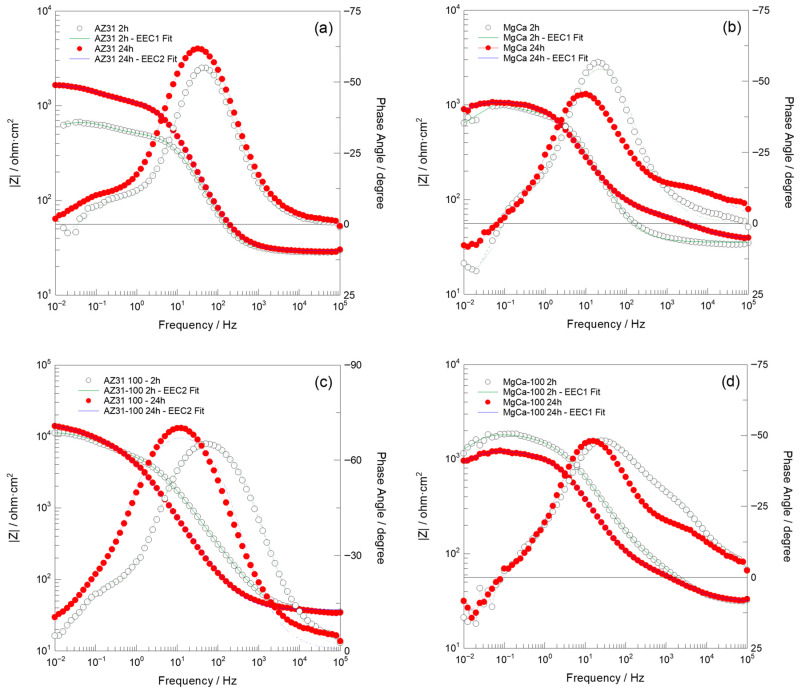
Bode plots recorded over the 100 kHz–10 mHz frequency range, along with fit results obtained using the EEC1 and EEC2 equivalent circuits shown in Figure 9, for (**a**) AZ31, (**b**) MgCa, (**c**) AZ31-100, and (**d**) MgCa-100 samples after 2 and 24 h of immersion in PBS solution.

**Figure 9:**
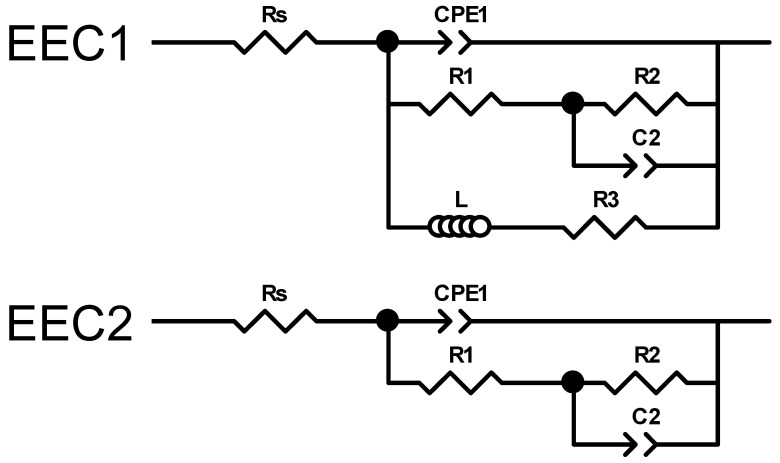
Electrical equivalent circuits used to assess the experimental impedance measurements.

Figure 7 and Figure 8 present the Nyquist and Bode impedance spectra, respectively, recorded over the 100 kHz–10 mHz frequency range, along with the fitting results obtained using the equivalent circuits EEC1 and EEC2 shown in Figure 9. These spectra correspond to the (a) AZ31, (b) MgCa, (c) AZ31-100, and (d) MgCa-100 samples after 2 and 24 h of immersion in PBS solution. EIS cannot be applied to partially coated samples (30% and 60%) because the electrical signal preferentially flows through exposed metallic regions, bypassing coated areas. As a result, the measurement would only represent uncoated zones, not the combined behaviour of coated and uncoated surfaces.

After 2 h of immersion, the Nyquist impedance plots generally exhibited two semicircles. Additionally, an inductive arc was observed in all samples except for the AZ31-100 coated sample. This sample displayed significantly higher impedance values compared to the uncoated samples and the MgCa-100 coated sample, suggesting superior barrier properties [106]. With prolonged immersion (24 h), the presence of two semicircles persisted, with increased diameters for AZ31 and MgCa, indicating improved corrosion performance over time. AZ31-100 maintained its superior behaviour with significantly larger semicircles [106]. However, the MgCa-100 sample exhibited a distinct behaviour, retaining the inductive loop observed during earlier immersion periods, which can be attributed to its high reactivity. The Bode plots (Figure 8) complement the Nyquist plots (Figure 7) by showing the frequency-dependent behaviour of the impedance modulus (∣Z∣) and phase angle. Higher ∣Z∣ values at low frequencies correlate with better corrosion protection properties. AZ31-100 consistently outperformed other samples, demonstrating enhanced barrier effectiveness against corrosion [107,108].

Due to the pronounced electrochemical reactivity of magnesium alloys, accurate interpretation of impedance spectra is a significant challenge, requiring careful selection of appropriate equivalent circuit models [109]. The equivalent circuit EEC1, shown in Figure 9, proposed by King et al. [110], provides a satisfactory fit for samples that exhibit two capacitive arcs and an inductive loop at the lowest frequencies. In contrast, the equivalent circuit EEC2, also shown in Figure 9, provides a reasonable fit result for impedance spectra that exhibit two capacitive arcs, when no inductive loop is present within the measured frequency range (100 kHz–10 mHz). This circuit has also been successfully used by Neves et al. [47,111] in studies on the corrosion behaviour of uncoated and coated Mg alloys with inorganic films for biomedical applications.

It is worth noting that a detailed explanation of the physical meaning of each circuit element is beyond this paper’s scope. This topic remains under active scientific debate and is currently under intensive investigation [112,113]. Gomes et al. [114] reported that for uncoated Mg electrodes, the capacitive arc observed in the high-frequency region was associated with the charge transfer resistance in parallel with the interfacial capacitance. The second arc, identified at intermediate frequencies, was ascribed to the diffusion of electroactive species through the corrosion products. Finally, the inductive arc detected in the low-frequency domain was attributed to the relaxation of adsorbed intermediates on the magnesium surface. Conversely, Neves et al. [111] observed that for uncoated Mg alloys, the time constant at high frequencies was of a magnitude typically associated with a thin oxide layer, which was modelled using a constant phase element (CPEₒₓ) in parallel with an oxide resistance (Rₒₓ). The intermediate time constant was ascribed to ongoing electrochemical activity (corrosion processes) and was modelled as a constant phase element (CPE_dl_) in parallel with the charge transfer resistance (Rct). Notably, these authors did not utilize the low-frequency data to extract information regarding inductive behaviour in these Mg alloys, which contrast to the studies reported by King et al. [110].

The results obtained in this study are more consistent with those reported by Neves et al. [47,111]. In particular, Table A15 and Table A16 present the calculated values of each electrical element in the equivalent circuits (EECs) shown in Figure 9, together with the error percentages, minimum weighted sum of squares (WSS), and excellent chi-squared (χ^2^) values (~10^−3^–10^−4^) [106]. These results, obtained by fitting the experimental impedance spectra of AZ31, MgCa, AZ31-100, and MgCa-100 after 2 and 24 h of immersion in PBS solution using ZView software, demonstrate a highly satisfactory agreement with the experimental data. Consistently, the fit plots generated through this fitting process further support this conclusion, as the Nyquist and Bode fit spectra exhibit a close match with the experimental impedance responses (Figure 7 and Figure 8).

Consistent with previous studies, the results suggest that the high- and middle-frequency regions of the impedance spectra for the uncoated AZ31 and MgCa samples are influenced by the electrical resistance of passive films or native oxide layers spontaneously formed on the magnesium alloy surfaces (R_ox_) [115], as well as by the charge transfer resistance at the metal/electrolyte interface (R_ct_) [116], or a combination of both [117,118,119,120]. In contrast, in the coated AZ31-100 and MgCa-100 samples, the high-frequency region of the impedance spectra is additionally influenced by the presence of HMZ coatings. This observation is consistent with previous reports on the corrosion protection behaviour of sol–gel-coated magnesium alloys in aqueous environments [106,119], further supporting the influence of surface modification on the electrochemical response.

In complex scenarios such as the present study, where the comparative assessment of corrosion and degradation behaviour among alloys and coatings with varying compositions is required, the low-frequency impedance modulus (|Z|_0.01Hz_) is widely recognized as a reliable and practical parameter for evaluation [107,108,121,122]. Table 2 present the Impedance modulus (|Z|) at low-frequency (f = 0.01 Hz) and the cumulative electrical resistance values (R_sum_), where R_sum_ = Rs + R1 + R2, determined by fitting the experimental impedance spectra to the EEC1 and EEC2 equivalent circuits, obtained after 2 and 24 h of immersion of the magnesium alloy samples and in PBS solution. In general, a higher impedance value in the low-frequency region indicates better barrier properties and results in the improvement of the corrosion protection of the coating [121,122].

The results presented in Table 2 show that after 2 h of immersion in PBS, the corrosion protection performance of each of the tested samples is ranked as follows: AZ31-100 >> MgCa-100 > MgCa > AZ31. After 24 h, this order is as follows: AZ31-100 >> MgCa-100 > AZ31 > MgCa. Slight discrepancies in |Z|_0.01Hz_ and R_sum_ values (Table 2) are related to the influence of the inductive arc resistance on the |Z|_0.01Hz_ measurements. These results indicate that the coating on AZ31 provides the best corrosion protection, which is especially relevant given its highly corrodible surface. Conversely, MgCa alloys exhibited diminished effectiveness in terms of corrosion protection compared to AZ31 alloys, even when coated. A 24 h immersion slightly improves the corrosion resistance of uncoated samples, likely due to salt deposition from their own dissolution. However, in the coated samples, corrosion resistance remains virtually unchanged for MgCa-100, or even worsens for AZ31-100, likely due to coating degradation over time. In this case, the degradation outweighs any additional protection against dissolution byproducts, unlike what happens on uncoated surfaces.

EIS has proven highly effective for evaluating fully coated and uncoated magnesium alloys; however, its intrinsic limitations prevent accurate assessment of partially coated surfaces. Future studies will allow us to incorporate localized techniques such as LEIS and LEIM to achieve spatially resolved characterization of heterogeneous coatings.

### 3.2. Response to Microorganisms of the Electrodeposited Coating

#### 3.2.1. Action on Sessile Bacteria

**Figure 10:**
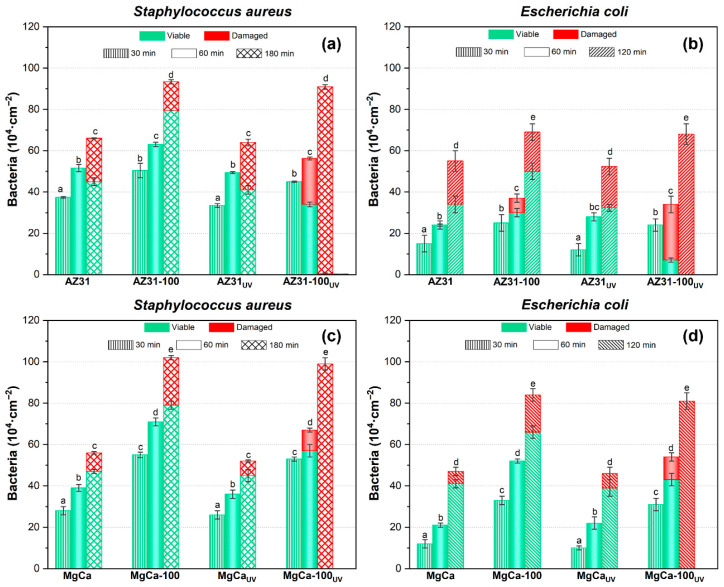
Areal density of adhered bacteria and viability of *S. aureus* and *E. coli* on AZ31 (**a**,**b**) and MgCa (**c**,**d**) surfaces, before and after ultraviolet light exposure. The times shown correspond to bacterial contact periods. Green and red bars represent viable and damaged bacteria, respectively; error bars correspond to mean ± standard deviation (*n* = 3). Different letters among the data indicate statistically significant differences in the total number of adhered bacteria (*p* < 0.05) among all tested conditions within each bacterial species (coated, uncoated, UV-irradiated, and non-irradiated samples). Statistical comparisons were applied only to total areal bacterial density, while colour distributions represent descriptive data with their corresponding variability. Statistical tests performed in this experiment are included in Appendix A.

Figure 10 shows the areal density of adhered *S. aureus* and *E. coli* on AZ31 (a, b) and MgCa (c, d) alloys, both coated and uncoated, after different contact times between the bacterial suspensions and the surfaces. All UV-activated samples were irradiated for 24 h prior to bacterial deposition. The times indicated in Figure 10 correspond exclusively to the bacterial contact period on the irradiated surface, after completion of the UV treatment, as described in the Materials and Methods section. Figure 10 also includes the results obtained on the same surfaces after ultraviolet irradiation prior to bacterial exposure. For all surfaces and both bacterial species, the areal density of adhered bacteria increased with contact time. UV pre-treatment of the samples did not significantly affect bacterial adhesion for either strain on any surface. Coated surfaces exhibited higher bacterial adhesion than uncoated ones. *S. aureus* showed a greater adhesion capacity than *E. coli*, particularly on coated surfaces. On non-irradiated samples, damaged bacteria were observed after 180 min of contact for *S. aureus* and 120 min for *E. coli*, except on AZ31-100, where some *E. coli* appeared damaged as early as 60 min after contact. For UV-irradiated surfaces, the bactericidal effect of the uncoated alloys was similar to that observed without irradiation. In contrast, UV irradiation of coated surfaces activated their antibacterial properties, resulting in more efficient bacterial damage. On UV-irradiated coated samples, damaged *S. aureus* and *E. coli* cells were detected after 60 min of contact. The effect was more pronounced for *E. coli* on AZ31-100. Complete bacterial damage was observed after 180 min for *S. aureus* and 120 min for *E. coli*. Statistically significant differences were found (*p* < 0.05).

The effect of the coatings on bacterial damage, according to Equation (1), was quantified by calculating the percentage of damaged bacteria (%D) relative to the total number of adhered bacteria on each surface and at each contact time (Table 3). A colour scale is used in Table 3, where darker red indicates a higher %D, facilitating data visualization and interpretation. On MgCa surfaces—both coated and uncoated, and either UV-irradiated or not—a limited antibacterial activity was observed after 120 min of contact, with %D ranging from 10% to 25%, except on the fully coated and UV-irradiated surface that reached 99 ± 1% of damage. AZ31-based surfaces initially exhibited higher %D values compared to MgCa-based ones. Both irradiated and non-irradiated AZ31 surfaces produced moderate bacterial damage, with %D values ranging between 30% and 40%. Differences between the base alloys disappeared after UV irradiation of the coated surfaces. Under these conditions, both coatings showed comparable antibacterial performance, achieving nearly complete bacterial damage after 180 min for *S. aureus* and 120 min for *E. coli*.

The previous results, based on the Live/Dead assay, only indicate that bacterial cells were damaged, but do not confirm their loss of viability. However, serial dilution and plate culture methods provide more reliable information regarding bacterial viability. In these methods, microorganisms are allowed to grow in nutrient-rich media after contact with the tested surfaces. Viable cells can be recovered if the inflicted damage was not sufficient to inactivate them. Table 4 presents the antibacterial rate (%AR) evaluated according to Equation (2) for all tested surfaces, including coatings with 30% and 60% surface coverage. The values correspond to the contact time that produced the highest level of bacterial damage. A colour scale is used in Table 4, where darker red indicates a higher %AR, facilitating data interpretation. UV-irradiated AZ31 surfaces with 100% and 60% coating coverage exhibited %AR values close to 99% for both *S. aureus* and *E. coli*.

A similar trend was observed for MgCa surfaces, although slightly lower efficacy was found against *E. coli*. For coatings with only 30% surface coverage, %AR values were lower than those observed in fully coated samples but still higher than in the uncoated alloys. Bacterial viability on non-irradiated surfaces was significantly higher than on irradiated ones, with no notable differences between coated and uncoated samples at any coverage level—except for *E. coli* on MgCa.

#### 3.2.2. Action on Planktonic Bacteria

Magnesium alloys and their coatings degrade when immersed in aqueous media. The degradation products can alter the surrounding environment, potentially affecting the viability of nearby planktonic bacteria, even if adhesion to the surface does not occur. Table 5 presents the antibacterial rate (%AR). A colour scale is used, where darker red indicates higher %AR values, facilitating data interpretation for planktonic bacteria exposed to the surfaces during two specific time periods: (1) the contact time required to reach ~99% AR in sessile bacteria (180 min for *S. aureus* and 120 min for *E. coli*), and (2) extended contact periods during which %AR for planktonic bacteria also approached 99% (300 min for *S. aureus* and 180 min for *E. coli*). The %AR trend for planktonic bacteria generally followed the same pattern as observed for sessile bacteria, but with slightly lower efficacy at the same contact times. The highest %AR values observed for AZ31-100_UV_ and AZ31-60_UV_ after 180 min were approximately 60% for *S. aureus* and 70% for *E. coli*. For MgCa-100_UV_ and MgCa-60_UV_ after 120 min, values ranged between 57% and 73% for *S. aureus*, and between 44% and 62% for *E. coli*. As observed for sessile bacteria, all non-irradiated surfaces exhibited low %AR values against both bacterial strains.

#### 3.2.3. Action on Bacterial Biofilms

The results of biofilm production (%B), evaluated through the ATP production of microorganisms embedded in a 24 h mature biofilm, for all surfaces and both bacterial species, are listed in Table 6. Positive %B values, indicating biofilm reduction, are shown in red, while negative %B values, representing increased biofilm formation, are marked in green. For the %B analysis, untreated AZ31 and MgCa surfaces were used as controls. All coated surfaces that were not UV-irradiated showed increased biofilm formation for both *S. aureus* and *E. coli*, regardless of alloy type or coating extent. However, significant biofilm reduction was observed on all surfaces when they were UV-irradiated prior to biofilm formation. This reduction was more pronounced in the samples with the highest electrodeposited surface coverage.

## 4. Discussion

Implantable devices may suffer surface damage during surgical placement [79,80]. This can compromise coating integrity and leave unprotected areas [19,81] exposed to bacterial colonization. This study investigates how partial coating loss affects bacterial colonization. AZ31 and MgCa magnesium alloys coated with HMZ were used for this purpose.

Uncoated AZ31 surfaces showed lower roughness and fewer topographic features than MgCa (Table 1). Electrically, both alloys exhibited a positive zeta potential, although lower in AZ31. Since *S. aureus* and *E. coli* carry a net negative surface charge in physiological media [123], higher adhesion would be expected on more positively charged surfaces, such as MgCa. However, the higher bacterial adhesion observed on AZ31 suggests that surface chemistry has a greater influence than electrostatic interactions.

XPS and XRD analyses confirmed the presence of hydroxyapatite, magnesium phosphate, and zinc oxide on the electrodeposited surfaces. This confirms that ZnO formation mainly occurred during the alkaline post-treatment, consistent with the dehydration of Zn(OH)_2_ and the concurrent crystallization of hydroxyapatite under alkaline conditions. These compounds are widely recognized for their bioactive properties [8,45,46,48,124,125,126,127,128,129]. Although the electrodeposited composition was identical in both alloys, the coated surfaces behaved differently from their respective uncoated counterparts. Electrodeposition significantly increased roughness parameters such as Sp, Sq, and Sdr. However, after coating, both alloys displayed a comparable surface morphology—more homogeneous and isotropic. Both materials also exhibited a shift toward a more negative surface charge. The zeta potential of coated AZ31 became negative, while that of coated MgCa decreased compared to uncoated MgCa but remained positive. This change in zeta potential is attributed to the combined effect of the electrodeposition and subsequent alkaline treatment, which increases surface hydroxylation. This behaviour may explain the higher bacterial colonization observed on coated MgCa compared to coated AZ31 for both negatively charged bacterial strains [123].

Bacterial adhesion increased more rapidly on coated MgCa, even at the shortest contact time evaluated. It is worth noting that bacterial morphology may also influence adhesion. *S. aureus*, with a spherical shape, appears to adapt better to surface topography than *E. coli*, which has an elongated shape. This may explain why *E. coli* adhesion was lower compared to *S. aureus*. Nevertheless, these adhesion patterns were not affected by UV irradiation, either on coated or uncoated surfaces.

Beyond bacterial adhesion, the bactericidal capacity of the surfaces was evaluated using two complementary approaches: viability staining (%D) and serial dilution plating (%AR). Viability staining revealed high levels of damaged bacteria (>98%) only after prolonged contact. This occurred at 180 min for *S. aureus* and 120 min for *E. coli* on UV-irradiated coated surfaces of both alloys. However, this technique only indicates membrane integrity loss and does not confirm cell death. This strong antibacterial effect is consistent with the well-documented photocatalytic behaviour of ZnO under UV irradiation [45,67,68,69,70,71,72,73,74,75,76,77,78,130]. ZnO is an n-type semiconductor with a band gap comparable to that of TiO_2,_ enabling surface photoactivation. Upon UV exposure, photons with energy above its band gap excite electrons from the valence to the conduction band, generating electron-hole pairs. These charge carriers react with water and oxygen to produce hydroxyl radicals (·OH) and superoxide anions ($O_{2}^{-}\cdot$), which induce oxidative stress, membrane disruption and cell death. Although reactive oxygen species (ROS) were not directly quantified in this study, the pronounced bactericidal response observed after UV exposure supports the hypothesis of a surface-mediated oxidant mechanism. To determine bacterial viability, colony-forming units (CFU) were quantified from bacteria detached from the surfaces. Under these conditions, the antibacterial reduction percentage (%AR) confirmed that UV treatment over the full surface area induced a viability loss exceeding 98% for both *S. aureus* and *E. coli*, demonstrating a strong bactericidal effect upon coating activation.

In the absence of UV irradiation, none of the surfaces showed significant bactericidal activity. Only uncoated AZ31 exhibited some efficacy against *E. coli*, likely due to higher Mg^2+^ release and pH elevation during degradation [20,21,22,23,53,54,55,56,57,58,60,61,62]. This inherent antibacterial behaviour of magnesium is associated with Mg^2+^ ion release and the resulting local alkalisation during corrosion. In coated samples, this effect was strongly reduced due to the protective nature of the HMZ layer, indicating that the antibacterial response observed after UV activation originates from the photocatalytic ZnO rather than from substrate degradation. Although the electrodeposited coatings substantially improved corrosion resistance, they did not display antibacterial activity without UV activation. pH values and Mg^2+^ release were similar for irradiated and non-irradiated surfaces, indicating that the antibacterial effect observed after UV exposure results from the photocatalytic activity of ZnO and the generation of reactive oxygen species (ROS). No dissolved Zn^2+^ was detected under any condition. This agrees with the low solubility of ZnO in neutral or alkaline media and with the formation of insoluble Zn species such as Zn(OH)_2_ or zinc phosphates. These compounds immobilize Zn within the coating–solution interface, preventing ion release. Therefore, the antibacterial efficacy after UV irradiation is not related to Zn^2+^ release but to the photocatalytic activity.

In contrast, UV activation triggered a pronounced bactericidal effect on fully coated surfaces. After 180 min (*S. aureus*) or 120 min (*E. coli*), %D and %AR values exceeded 98%, indicating complete loss of bacterial viability. In comparison, uncoated surfaces maintained limited antibacterial activity, even after UV exposure.

Regarding planktonic bacteria, uncoated AZ31 and MgCa surfaces exhibited limited viability reduction after 180 min (*E. coli*) and 300 min (*S. aureus*), with %AR values not exceeding 31% for AZ31 and 40% for MgCa. Fully coated, non-irradiated surfaces (AZ31-100 and MgCa-100) showed even lower efficacy, with reductions never surpassing 35%.

UV activation markedly improved antibacterial activity in planktonic conditions as well. After 180 min (*E. coli*) or 300 min (*S. aureus*), AZ31-100_UV_ and MgCa-100_UV_ achieved %AR values above 96% for both strains. It is noteworthy that bacterial viability loss occurred faster in adhered bacteria than in planktonic cells, suggesting that UV-activated coatings exert a more immediate effect under direct contact conditions.

Biofilm formation was assessed by measuring bacterial metabolic activity after 24 h of incubation. Uncoated AZ31 and MgCa surfaces were used as reference values, with high levels of biofilm formation for both bacterial strains.

Without UV activation, fully coated surfaces (AZ31-100 and MgCa-100) did not reduce biofilm metabolic activity. In fact, they exhibited an increase compared to uncoated alloys. This behaviour suggests that the coatings may promote biofilm formation, possibly due to increased surface roughness and reduced ion release during the initial contact period.

In contrast, after UV activation, a significant reduction in biofilm metabolic activity was observed on fully coated surfaces. AZ31-100_UV_ showed a 73% reduction against *S. aureus* and 80% against *E. coli*, while MgCa-100_UV_ achieved even higher values, with reductions of 84% and 86%, respectively. These results indicate that the UV-activated coating effectively compromises the viability of bacteria embedded in biofilm, reinforcing its potential as an antibacterial strategy in clinical contexts where biofilm formation represents a major risk. This strong bactericidal action originates from surface-level interactions, as the antibacterial mechanism occurs at the outermost layer where bacteria directly contact the coating and the photoactivated ZnO.

Having established the strong antibacterial performance of fully coated surfaces, the next step was to determine whether this effect could be maintained in the event of partial coating loss during implantation. To this end, surfaces with controlled coverage on 30% and 60% of their area were analyzed to simulate defects that may arise during surgical procedures.

In the absence of UV irradiation, partially coated surfaces exhibited similar behaviour to fully coated but non-irradiated ones, with %AR values against adhered bacteria ranging from low to moderate. In AZ31, percentages remained around 30–45%, while in MgCa, they were slightly lower. These results suggest that, without UV activation, the percentage of coated area does not substantially affect bacterial viability on the surface.

However, after UV irradiation, the scenario changed significantly. Surfaces with 60% of coated area showed antibacterial responses nearly indistinguishable from fully coated area and irradiated surfaces, reaching %AR values close to 98% against both *S. aureus* and *E. coli*. This is particularly relevant, as it indicates that full surface coverage is not required to achieve robust bactericidal activity. Even with 30% coverage, substantial reductions were observed after UV activation—up to 63% in AZ31 against *E. coli* and 54% against *S. aureus*—demonstrating functional antibacterial performance under limited coverage conditions. Previous studies with different partial coating morphologies showed similar antibacterial responses.

For planktonic bacteria, it was previously shown that %AR values remained low without irradiation, and this trend was similar in partially coated surfaces. However, UV activation once again proved critical: 60% coated area surfaces reached %AR values between 95–96% in AZ31 and 85–86% in MgCa for both bacterial strains, comparable to fully coated area surfaces. Surfaces with 30% coated area also showed notable antibacterial activity after irradiation, with %AR values ranging from 61% to 74%, depending on the alloy and bacterial species.

This trend was confirmed in biofilm experiments as well. In the absence of UV activation, partially coated surfaces not only failed to reduce biofilm metabolic activity but, in some cases, even promoted biofilm compared to uncoated controls. However, after irradiation, 60% coated area surfaces showed biofilm reductions of 73% and 80% in AZ31 against *S. aureus* and *E. coli*, respectively, and 82–84% in MgCa—values nearly identical to those observed in fully coated area surfaces. Even with 30% area treatment, the effect remained relevant, with biofilm activity reductions ranging from 40% to 61%, confirming that UV-activated ZnO retains antibacterial effectiveness despite incomplete surface coverage.

Overall, these results demonstrate that the developed coating—composed of hydroxyapatite, magnesium phosphate, and zinc oxide—exhibits strong antibacterial activity when UV-activated, not only against adhered and planktonic bacteria but also against biofilms. The most significant finding of this study is that with only a 60% of the area treated is sufficient to replicate the antibacterial response of a fully coated surface, achieving over 95% viability reduction in all tested conditions. Even a more limited coated area, such as 30%, produced a moderate yet functional response after UV activation. These findings represent a major contribution to understanding the behaviour of implants against bacterial colonization when they undergo manipulation and potential surface damage during clinical procedures.

## 5. Conclusions

In this study, a functional coating composed of hydroxyapatite, magnesium phosphate, and zinc oxide was developed and applied via electrodeposition onto AZ31 and MgCa magnesium alloys. The coating significantly improved corrosion resistance without compromising the surface’s bioactive properties. In the absence of UV irradiation, the coated surfaces did not exhibit notable antibacterial activity against adhered bacteria, planktonic cells, or biofilm. However, after UV activation, a strong bactericidal effect was induced, with viability reductions exceeding 98% in all tested bacterial scenarios.

The most relevant finding of this work lies in demonstrating the efficacy of partially coated surfaces. A 60% surface coverage was sufficient to faithfully replicate the antibacterial performance of fully coated surfaces, even against biofilms. Moreover, 30% coated surfaces also exhibited moderate antibacterial activity after UV activation, indicating that functional effects are maintained even with limited coverage.

These results demonstrate that the UV-activated coating is not only effective against different bacterial colonization modes but also retains its antibacterial functionality despite partial loss—reinforcing its clinical applicability in real-world scenarios involving surface damage during implantation.

## Figures and Tables

**Table 1:** Quantitative data of Sq, Sp, Sv, Ssk, Sku, Str, and Sdr for each sample type at a 10× magnification.

	AZ31	MgCa	AZ31-100	MgCa-100
**Sq (µm) **	2.0 ± 0.4	2.3 ± 0.3	12 ± 2	13 ± 3
**Sp (µm)**	3.8 ± 0.3	4.2 ± 0.4	37 ± 5	35 ± 8
**Sv (µm)**	8.9 ± 1.2	9.3 ± 0.9	27 ± 7	31 ± 7
**Ssk**	−1.7 ± 0.3	−1.4 ± 0.3	0.6 ± 0.3	0.30 ± 0.18
**Sku**	7 ± 2	5.8 ± 1.4	3.1 ± 0.5	2.8 ± 0.4
**Str**	0.18 ± 0.11	0.14 ± 0.05	0.79 ± 0.09	0.74 ± 0.09
**Sdr (%)**	1.6 ± 0.6	2.1 ± 0.6	12.0 ± 1.2	7.8 ± 1.6

**Table 2:** Impedance modulus (|Z|) at low frequency (f = 0.01 Hz) and cumulative electrical resistance values (R_sum_), where R_sum_ = Rs + R1 + R2, determined by fitting the experimental impedance spectra to the EEC1 and EEC2 equivalent circuits shown in Figure 9 using ZView software. Immersion time: 2 h and 24 h.

	2 h		24 h	
**Sample**	**|Z|_0.01Hz_/Ω·cm^2^**	**R_sum_ (Ω·cm^2^)**	**|Z|_0.01Hz_/Ω·cm^2^**	**R_sum_ (Ω·cm^2^)**
AZ31	622.0 ± 3.6	712.9 ± 16.9	1659 ± 24	1663.4 ± 16.9
MgCa	643.6 ± 5.1	1087.4 ± 42.7	888.0 ± 10.9	1115.8 ± 7.8
AZ31-100	11,172 ± 68	11,634.8 ± 3.4	13,577 ± 104	10,528.6 ± 22.0
MgCa-100	1150 ± 16	1729.6 ± 20.6	961.3 ± 9.9	1800.8 ± 7.8

**Table 3:** Percentage of damaged *S. aureus* and *E. coli* bacteria relative to the number of adhered bacteria (%D) for AZ31, MgCa, AZ31-100, and MgCa-100 surfaces, with and without UV treatment, at different contact times (30, 60, and 180 min). Values are expressed as mean ± standard deviation of three independent biological replicates (*n* = 3), each obtained from two technical measurements.

*S. aureus* (%D)								
**Contact Time (min)**	**AZ31**	**AZ31_UV_**	**AZ31-100**	**AZ31-100_UV_**	**Mgca**	**MgCa_UV_**	**MgCa-100**	**MgCa-100_UV_**
30	0 ± 0	0 ± 0	0 ± 0	0 ± 0	0 ± 0	0 ± 0	0 ± 0	0 ± 0
60	0 ± 0	0 ± 0	0 ± 0	40 ± 1	0 ± 0	0 ± 0	0 ± 0	10 ± 2
180	32 ± 1	36 ± 2	15 ± 1	98 ± 2	16 ± 2	14 ± 3	23 ± 4	99 ± 1
***E. coli*** ** (%D)**								
Contact time (min)	AZ31	AZ31_UV_	AZ31-100	AZ31-100_UV_	MgCa	MgCa_UV_	MgCa-100	MgCa-100_UV_
30	0 ± 0	0 ± 0	0 ± 0	0 ± 0	0 ± 0	0 ± 0	0 ± 0	0 ± 0
60	0 ± 0	0 ± 0	19 ± 4	79 ± 2	0 ± 0	0 ± 0	0 ± 0	20 ± 3
120	38 ± 2	37 ± 3	28 ± 4	99 ± 1	13 ± 1	15 ± 2	21 ± 2	99 ± 1

**Table 4:** Antibacterial rate (%AR) of *S. aureus* and *E. coli* for AZ31 and MgCa surfaces and their coatings, with and without UV irradiation, at different contact times. Values are expressed as mean ± standard deviation of three independent biological replicates (*n* = 3), each obtained from two technical measurements.

*S. aureus* (%AR). Contact Time: 180 min							
AZ31	AZ31-30	AZ31-60	AZ31-100	MgCa	MgCa-30	MgCa-60	MgCa-100
38 ± 2	32 ± 1	30 ± 1	29 ± 1	27 ± 1	25 ± 1	25 ± 1	24 ± 1
**AZ31_UV_**	**AZ31-30_UV_**	**AZ31-60_UV_**	**AZ31-100_UV_**	**MgCa_UV_**	**MgCa-30_UV_**	**MgCa-60_UV_**	**MgCa-100_UV_**
38 ± 1	54 ± 2	99 ± 1	98 ± 2	28 ± 3	48 ± 2	99 ±1	98 ± 2
***E. coli*** ** (%AR). Contact time: 120 min**							
**AZ31**	**AZ31-30**	**AZ31-60**	**AZ31-100**	**MgCa**	**MgCa-30**	**MgCa-60**	**MgCa-100**
46 ± 2	45 ± 2	44 ± 1	44 ± 1	55 ± 2	38 ± 1	33 ± 2	32 ± 1
**AZ31_UV_**	**AZ31-30_UV_**	**AZ31-60_UV_**	**AZ31-100_UV_**	**MgCa_UV_**	**MgCa-30_UV_**	**MgCa-60_UV_**	**MgCa-100_UV_**
47 ± 1	63 ± 2	97 ± 2	98 ± 2	56 ± 1	53 ± 1	85 ± 4	90 ± 2

**Table 5:** Antibacterial rate (%AR) of planktonic *S. aureus* and *E. coli* near AZ31 and MgCa surfaces, with and without prior UV irradiation, at different contact times. Values are expressed as mean ± standard deviation of three independent biological replicates (*n* = 3), each obtained from two technical measurements.

*S. aureus* (%AR). Contact Time: 180 min							
AZ31	AZ31-30	AZ31-60	AZ31-100	MgCa	MgCa-30	MgCa-60	MgCa-100
22 ± 1	18 ± 2	17 ± 3	15 ± 1	16 ± 1	34 ± 2	37 ± 3	40 ± 2
**AZ31_UV_**	**AZ31-30_UV_**	**AZ31-60_UV_**	**AZ31-100_UV_**	**MgCa_UV_**	**MgCa-30_UV_**	**MgCa-60_UV_**	**MgCa-100_UV_**
23 ± 1	33 ± 3	60 ± 3	58 ± 1	17 ± 2	48 ± 2	57 ± 2	73 ± 2
***S. aureus*** ** (%AR). Contact time: 300 min**							
**AZ31**	**AZ31-30**	**AZ31-60**	**AZ31-100**	**MgCa**	**MgCa-30**	**MgCa-60**	**MgCa-100**
48 ± 1	37 ± 3	35 ± 2	19 ± 1	25 ± 3	55 ± 3	59 ± 3	64 ± 3
**AZ31_UV_**	**AZ31-30_UV_**	**AZ31-60_UV_**	**AZ31-100_UV_**	**MgCa_UV_**	**MgCa-30_UV_**	**MgCa-60_UV_**	**MgCa-100_UV_**
49 ± 1	74 ± 1	96 ± 3	96 ± 3	27 ± 2	61 ± 2	82 ± 3	97 ± 1
***E. coli*** ** (%AR). Contact time: 120 min**							
**AZ31**	**AZ31-30**	**AZ31-60**	**AZ31-100**	**MgCa**	**MgCa-30**	**MgCa-60**	**MgCa-100**
**31 ± 1**	28 ± 3	29 ± 2	29 ± 3	12 ± 1	14 ± 2	14 ± 3	14 ± 2
**AZ31_UV_**	**AZ31-30_UV_**	**AZ31-60_UV_**	**AZ31-100_UV_**	**MgCa_UV_**	**MgCa-30_UV_**	**MgCa-60_UV_**	**MgCa-100_UV_**
**31 ± 3**	65 ± 2	70 ± 1	74 ± 2	11 ± 2	31 ± 1	44 ± 2	62 ± 2
***E. coli*** ** (%AR). Contact time: 180 min**							
**AZ31**	**AZ31-30**	**AZ31-60**	**AZ31-100**	**MgCa**	**MgCa-30**	**MgCa-60**	**MgCa-100**
54 ± 1	43 ± 1	41 ± 1	40 ± 2	25 ± 3	52 ± 1	53 ± 3	53 ± 1
**AZ31_UV_**	**AZ31-30_UV_**	**AZ31-60_UV_**	**AZ31-100_UV_**	**MgCa_UV_**	**MgCa-30_UV_**	**MgCa-60_UV_**	**MgCa-100_UV_**
55 ± 1	74 ± 1	95 ± 3	97 ± 2	24 ± 2	61 ± 3	86 ± 2	98 ± 2

**Table 6:** Relative biofilm production percentages (%B) of *S. aureus* and *E. coli* on AZ31 and MgCa surfaces with different coatings, before and after UV irradiation, after 24 h of contact. Values are expressed as mean ± standard deviation of three independent biological replicates (*n* = 3), each obtained from two technical measurements.

*S. aureus* (%B). Contact Time: 24 h							
AZ31	AZ31-30	AZ31-60	AZ31-100	MgCa	MgCa-30	MgCa-60	MgCa-100
0 ± 1	−24 ± 4	−59 ± 3	−68 ± 2	0 ± 0	−26 ± 5	−73 ± 6	−83 ± 10
**AZ31_UV_**	**AZ31-30_UV_**	**AZ31-60_UV_**	**AZ31-100_UV_**	**MgCa_UV_**	**MgCa-30_UV_**	**MgCa-60_UV_**	**MgCa-100_UV_**
1 ± 1	30 ± 2	40 ± 2	73 ± 4	2 ± 2	45 ± 1	61 ± 4	84 ± 9
***E. coli*** ** (%B). Contact time: 24 h**							
**AZ31**	**AZ31-30**	**AZ31-60**	**AZ31-100**	**MgCa**	**MgCa-30**	**MgCa-60**	**MgCa-100**
0 ± 1	−46 ± 1	−78 ± 2	−106 ± 6	0 ± 1	−91 ± 3	−127 ± 13	−158 ± 7
**AZ31_UV_**	**AZ31-30_UV_**	**AZ31-60_UV_**	**AZ31-100_UV_**	**MgCa_UV_**	**MgCa-30_UV_**	**MgCa-60_UV_**	**MgCa-100_UV_**
−4 ± 1	29 ± 2	59 ± 1	93 ± 2	1 ± 1	15 ± 5	74 ± 6	89 ± 4

## Data Availability

The original contributions presented in the study are included in the article, further inquiries can be directed to the corresponding author.

## Abbreviations

The following abbreviations are used in this manuscript:

HMZ	Coating composed of hydroxyapatite, magnesium phosphate and zinc oxide
AZ31	Magnesium alloy (AZ31B). Mg-3Al-1Zn
MgCa	Magnesium alloy (Mg0.6Ca). Mg-0.6Ca
AZ31-100	Full surface treatment on AZ31
MgCa-100	Full surface treatment on MgCa
AZ31-60	Partial surface treatment, 60% on AZ31
MgCa-60	Partial surface treatment, 60% on MgCa
AZ31-30	Partial surface treatment, 30% on AZ31
MgCa-30	Partial surface treatment, 30% on MgCa
UV	Ultraviolet light
ROS	Reactive oxygen species

## Appendix A

### Appendix A.1. XPS: Deconvolution of High-Resolution Spectra for C1s, O1s, and Ca2p Levels

The deconvolution of the high-resolution spectra for the C1s level of the AZ31 coating and MgCa coating, shown in Figure A1a and Figure A1b, respectively, identifies three main peaks at 285.1 eV, 289.9 eV, and 283.8 eV. These correspond to C–C/C–H, O–C=O, and C-metal bonds [131,132], indicative of typical environmental contamination encountered during sample handling.

Regarding the O1s level, the analysis for the AZ31 coating, detailed in Figure A1c, shows two peaks at 530.6 eV and 532.0 eV. Similarly, the MgCa coating, shown in Figure A1d, presents peaks at 530.5 eV and 529.2 eV. These binding energies, close to 530 eV, are commonly associated with phosphate and hydroxide groups in hydroxyapatite structures, O-metal bonds, and adsorbed carbonates [133,134]. Some studies also link these values to Mg_3_(PO_4_)_2_; [135] and ZnO [136,137]. The O1s peak at 532.0 eV is specifically attributed to oxygen in magnesium oxide (MgO) [135,138].

High-resolution spectra in the Ca2p energy region were also recorded, shown in Figure A1e for the AZ31 coating and Figure A1f for the MgCa coating. However, the overlapping kinetic energy of Mg KLL Auger electrons prevents detailed information about the calcium environment. Nevertheless, the significant contribution of these electrons indicates that the coating surfaces are magnesium-rich.

### Appendix A.2. ANOVA Analysis for Topography Measurements

ANOVA test can be implemented to obtain the conclusions in terms of topographical properties by 3D-roughness parameters set of the different Mg alloys surfaces before and after electrodeposition treatment. To understand better the information provided by ANOVA test, Table A1 and Table A2 recover all the main statistical parameters.

**Table A1:** Brief description of the explained and explanatory variables, with their corresponding levels, considered in the experimental statistical design.

Explained Variable (Response)	Explanatory Variables (Factors)	Levels (Code)
3D-Roughness Parameter	Type of Mg alloy	AZ31
		MgCa
	Electrodeposition Treatment	Uncoated
		Coated

To test for the existence of significant differences in the values of the 3D roughness parameter, a two-way analysis of variance (ANOVA) and subsequent Tukey’s multiple comparison were performed. In this case, the factors (explanatory variables) are alloy type and surface treatment, while the response variable (explained variable) is the evaluated parameter. The statistical *p*-values of the different analyses on each parameter are shown in Table A2 for 10× magnification.

**Table A2:** Summary of the *p*-values of the two-way ANOVA on each 3D roughness parameter in the comparison between alloys and surface treatments (at 95% confidence level), for 10× magnification.

*p* Values Two-Way ANOVA	3D Roughness Parameter								
	Sq	Ssk	Sku	Sp	Sv	Sz	Sa	Str	Sdr
Alloy	0.6455	0.4306	0.0047 *	0.2700	0.0299 *	0.4614	0.6090	0.0314 *	<0.0001 ***
Treatment	<0.0001 ***	<0.0001 ***	<0.0001 ***	<0.0001 ***	<0.0001 ***	<0.0001 *	<0.0001 **	<0.0001 **	<0.0001 ***
Interaction	0.7108	<0.0001 ***	0.0866	0.1277	0.0734	0.7895	0.6962	0.7945	<0.0001 ***

* *p* ≤ 0.05, ** *p* ≤ 0.01, *** *p* ≤ 0.001.

Similarly, a two-way analysis of variance (ANOVA) and subsequent Tukey’s multiple comparison were performed on the 3D roughness parameter values obtained at 20× magnification. The *p*-statistic values of the different analyses on each parameter are shown in Table A3.

**Table A3:** Summary of the *p*-values of the two-way ANOVA on each 3D roughness parameter in the comparison between alloys and surface treatments (at 95% confidence level), for 20× magnification.

*p* Values Two-Way ANOVA	3D Roughness Parameter								
	Sq	Ssk	Sku	Sp	Sv	Sz	Sa	Str	Sdr
**Alloy**	0.2649	0.0532	0.6894	0.2881	0.4436	0.6419	0.2397	0.0854 *	<0.0001 ***
**Treatment**	<0.0001 ***	<0.0001 ***	<0.0001 ***	<0.0001 ***	<0.0001 ***	<0.0001 *	<0.0001 **	<0.0001 **	<0.0001 ***
**Interaction**	0.3370	0.0063 **	0.1741	0.2736	0.2958	0.7362	0.3232	0.4971	<0.0001 ***

* *p* ≤ 0.05, ** *p* ≤ 0.01, *** *p* ≤ 0.001.

Again, a two-way analysis of variance (ANOVA) and subsequent Tukey’s multiple comparison were performed on the 3D roughness parameter values obtained at 50× magnification. The statistic *p*-values of the different analyses for each parameter are shown in Table A4.

**Table A4:** Summary of the *p*-values of the two-way ANOVA on each 3D roughness parameter in the comparison between alloys and surface treatments (at 95% confidence level), for the 50× magnification.

*p* Values Two-Way ANOVA	3D Roughness Parameter								
	Sq	Ssk	Sku	Sp	Sv	Sz	Sa	Str	Sdr
**Alloy**	0.2486	0.7295	0.3968	0.1726	0.0792	0.0798	0.3136	0.9192	<0.0001 ***
**Treatment**	<0.0001 ***	<0.0001 ***	<0.0001 ***	<0.0001 ***	<0.0001 ***	<0.0001 *	<0.0001 **	<0.0001 **	<0.0001 ***
**Interaction**	0.2472	0.2645	0.5976	0.1729	0.0806	0.0805	0.3141	0.0253 *	<0.0001 ***

* *p* ≤ 0.05, ** *p* ≤ 0.01, *** *p* ≤ 0.001.

**Table A5:** Summary of the statistical *p*-values for Sa and ANOVA residuals data after checking the normality and homocedasticity corresponding tests, as an example of the procedure for the remain 3D-roughness parameters.

Data Studied	Normality *p*-Value (Kolmogorov-Smirnov Test)	Homocedasticity *p*-Value (Bartlett Test)
Sa	0.14577	0.10254
Residuals from ANOVA	0.09748	0.10254

Hence, the ANOVA modelled test applied is hereby validated since all the conditions needed are fullfilled, i.e.,:

1. The independence of observations is maintained since the roughness parameters measurements had been performed in aleatory replicates of each alloy and surface treatment condition. Moreover, the measurements were also carried out in aleatory zones of each replicate sample. Any procedure pattern has been avoided through the entire experimental study and parameter acquisition.
2. The normality of the residuals obtained from the ANOVA model has been proved since the Kolmogorov-Smirnov test applied gives a *p*-value higher than the 5% of confidence, due to all analyses were considered at a significance level of *p*-value < 0.05. Hence, the null hypothesis, previously stablished as the existence of normality in the data, can not be rejected. This information has also been checked by QQ plots of the residual data.
3. The homocedasticity of the roughness parameters variances has been proved since the Bartlett test gives a *p*-value higher than the 5% of confidence, due to all analyses were considered at a significance level of *p*-value < 0.05. Hence, the null hypothesis, previously stablished as the existence of homocedasticity in the variances, can not be rejected.

In consequence, all the conclusions drawn from this ANOVA test described, in terms of the topographical properties evaluation, are considered supported by statistical confirmation.

### Appendix A.3. Quantitative Data for All the 3D-Roughness Parameters for Each Scanned Area

**Table A6:** Quantitative Sq data for each sample type and magnification.

Sq (µm) Magnification	AZ31	AZ31-100	MgCa	MgCa-100
**10×**	1.959 ± 0.379	12.499 ± 2.154	2.275 ± 0.349	12.533 ± 2.568
**20×**	0.562 ± 0.058	10.698 ± 1.676	0.515 ± 0.029	10.077 ± 2.063
**50×**	0.143 ± 0.007	7.785 ± 2.635	0.145 ± 0.008	6.999 ± 1.470

**Table A7:** Quantitative Ssk data for each sample type and magnification.

Ssk Magnification	AZ31	AZ31-100	MgCa	MgCa-100
**10×**	−1.663 ± 0.347	0.629 ± 0.254	−1.423 ± 0.261	0.295 ± 0.177
**20×**	−0.683 ± 0.138	0.462 ± 0.324	−0.632 ± 0.204	0.174 ± 0.324
**50×**	−0.658 ± 0.134	0.524 ± 0.373	−0.597 ± 0.208	0.409 ± 0.538

**Table A8:** Quantitative Sku data for each sample type and magnification.

Sku Magnification	AZ31	AZ31-100	MgCa	MgCa-100
**10×**	7.202 ± 2.220	3.134 ± 0.526	5.784 ± 1.448	2.774 ± 0.360
**20×**	3.627 ± 0.439	2.775 ± 0.438	3.823 ± 0.624	2.668 ± 0.442
**50×**	3.400 ± 0.427	2.987 ± 0.774	3.357 ± 0.414	2.805 ± 0.678

**Table A9:** Quantitative Sp data for each sample type and magnification.

Sp (µm) Magnification	AZ31	AZ31-100	MgCa	MgCa-100
**10×**	3.775 ± 0.324	36.693 ± 4.602	4.207 ± 0.447	34.693 ± 7.713
**20×**	1.140 ± 0.102	27.342 ± 4.970	1.173 ± 0.061	25.177 ± 7.400
**50×**	0.316 ± 0.027	21.431 ± 6.095	0.315 ± 0.032	18.836 ± 5.826

**Table A10:** Quantitative Sv data for each sample type and magnification.

Sv (µm) Magnification	AZ31	AZ31-100	MgCa	MgCa-100
**10×**	8.876 ± 1.221	26.874 ± 7.178	9.326 ± 0.902	31.424 ± 6.941
**20×**	1.960 ± 0.233	23.340 ± 4.533	1.774 ± 0.211	24.545 ± 3.778
**50×**	0.473 ± 0.048	16.708 ± 4.837	0.467 ± 0.048	14.360 ± 3.405

**Table A11:** Quantitative Sz data for each sample type and magnification.

Sz (µm) Magnification	AZ31	AZ31-100	MgCa	MgCa-100
**10×**	12.651 ± 1.357	63.567 ± 9.192	13.532 ± 1.357	65.449 ± 13.817
**20×**	3.101 ± 0.311	50.683 ± 6.079	2.947 ± 0.233	49.721 ± 8.774
**50×**	0.789 ± 0.060	38.139 ± 9.760	0.783 ± 0.057	33.196 ± 7.752

**Table A12:** Quantitative Sa data for each sample type and magnification.

Sa (µm) Magnification	AZ31	AZ31-100	MgCa	MgCa-100
**10×**	1.365 ± 0.306	10.099 ± 1.984	1.657 ± 0.288	10.138 ± 2.043
**20×**	0.440 ± 0.047	8.741 ± 1.552	0.393 ± 0.021	8.205 ± 1.556
**50×**	0.113 ± 0.004	6.302 ± 2.339	0.113 ± 0.006	5.709 ± 1.165

**Table A13:** Quantitative Str data for each sample type and magnification.

Str Magnification	AZ31	AZ31-100	MgCa	MgCa-100
**10×**	0.176 ± 0.111	0.792 ± 0.094	0.137 ± 0.046	0.742 ± 0.095
**20×**	0.211 ± 0.106	0.714 ± 0.141	0.131 ± 0.081	0.679 ± 0.223
**50×**	0.711 ± 0.083	0.642 ± 0.120	0.768 ± 0.104	0.580 ± 0.147

**Table A14:** Quantitative Sdr data for each sample type and magnification.

Sdr (%) Magnification	AZ31	AZ31-100	MgCa	MgCa-100
**10×**	1.635 ± 0.593	12.018 ± 1.183	2.056 ± 0.590	7.763 ± 1.594
**20×**	0.437 ± 0.106	24.714 ± 2.588	0.369 ± 0.036	12.652 ± 2.464
**50×**	0.084 ± 0.015	38.293 ± 9.953	0.0747 ± 0.014	24.416 ± 5.295

**Table A15:** Calculated values of each electrical element in the equivalent circuits (EECs) shown in Figure 9, along with error percentages, chi-squared (χ^2^) values, and weighted sum of squares (WSS), obtained by fitting the experimental impedance spectra of AZ31, MgCa, AZ31-100, and MgCa-100 samples after 2 h of immersion in PBS solution.

	Samples							
	AZ31		MgCa		AZ31-100		MgCa-100	
Electrical Elements of the Equivalent Circuits	EEC1		EEC1		EEC2		EEC1	
	Value	Error %	Value	Error %	Value	Error %	Value	Error %
**Rs/Ω·cm^2^**	28.89	0.32	35.43	0.75	34.81	0.44	29.46	1.11
**CPE1-T/F × s^α−1^ × cm^−2^**	5.65 × 10^−5^	2.04	9.45 × 10^−5^	3.48	1.88 × 10^−5^	1.21	6.26 × 10^−5^	14.36
**CPE1-P**	0.88	0.37	0.83	0.76	0.80	0.22	0.65	2.02
**R1/Ω × cm^2^**	4.87 × 10^2^	1.17	8.75 × 10^2^	2.09	6.26 × 10^3^	1.36	2.01 × 10^1^	20.47
**R2/Ω × cm^2^**	1.97 × 10^2^	16.88	1.77 × 10^2^	42.64	5.34 × 10^3^	3.13	1.68 × 10^3^	1.85
**CPE2-T/F × s^α−1^ × cm^−2^**	5.01 × 10^−3^	6.89	6.56 × 10^−3^	23.54	2.85 × 10^−4^	4.51	1.43 × 10^−5^	40.29
**CPE2-P**	0.81	8.87	1.07	18.86	0.82	2.87	0.85	6.38
**R3/Ω × cm^2^**	3.55 × 10^3^	17.91	1.12 × 10^3^	6.91	-	-	2.49 × 10^3^	5.56
**L/H × cm^−2^**	3.25 × 10^4^	36.17	1.15 × 10^4^	15.93	-	-	3.22 × 10^4^	5.23
**χ^2^**	2.17 × 10^−4^		1.25 × 10^−3^		2.63 × 10^−4^		7.28 × 10^−3^	
**WSS**	2.76 × 10^−2^		1.58 × 10^−1^		3.56 × 10^−2^		9.69 × 10^−2^	

**Table A16:** Calculated values of each electrical element in the equivalent circuits (EECs) shown in Figure 9, along with error percentages, chi-squared (χ^2^) values, and weighted sum of squares (WSS), obtained by fitting the experimental impedance spectra of AZ31, MgCa, AZ31-100, and MgCa-100 samples after 24 h of immersion in PBS solution.

	Samples							
	AZ31		MgCa		AZ31-100		MgCa-100	
Electrical Elements of the Equivalent Circuits	EEC2		EEC1		EEC2		EEC1	
	Value	Error %	Value	Error %	Value	Error %	Value	Error %
**Rs/Ω × cm^2^**	29.41	0.32	37.37	1.27	37.57	1.08	30.76	0.80
**CPE1-T/F × s^α−1^ × cm^−2^**	5.12 × 10^−5^	2.04	5.15 × 10^−5^	30.35	4.56 × 10^−5^	2.68	5.13 × 10^−5^	19.33
**CPE1-P**	0.87	0.37	0.65	4.51	0.82	0.61	0.68	2.71
**R1/Ω × cm^2^**	1.13 × 10^3^	1.17	4.84 × 10^1^	7.71	9.97 × 10^3^	5.03	6.20 × 10^2^	7.80
**R2/Ω × cm^2^**	5.04 × 10^2^	16.88	1.03 × 10^3^	0.75	5.21 × 10^2^	21.38	1.15 × 10^3^	0.54
**CPE2-T/F × s^α−1^ × cm^−2^**	3.05 × 10^−3^	5.62	1.04 × 10^−4^	11.20	6.81 × 10^−4^	36.52	5.71 × 10^−5^	12.76
**CPE2-P**	0.81	3.50	0.81	1.87	0.84	16.85	0.82	1.96
**R3/Ω × cm^2^**	-	-	2.72 × 10^3^	5.91	-	-	2.20 × 10^3^	3.97
**L/H × cm^−2^**	-	-	3.37 × 10^4^	6.19	-	-	2.97 × 10^4^	3.75
**χ^2^**	1.84 × 10^−4^		3.24 × 10^−3^		2.23 × 10^−3^		2.21 × 10^−4^	
**WSS**	2.50 × 10^−2^		4.31 × 10^−1^		3.18 × 10^−1^		2.94 × 10^−3^	

### Appendix A.4. ANOVA Analysis for Early Bacterial Adhesion Measurements

ANOVA test can be implemented to obtain the conclusions in terms of early bacterial adhesion on the different Mg alloys surfaces before and after electrodeposition and UV-irradiation treatments. To understand better the information provided by ANOVA test, Table A17 recovers all the main statistical parameters.

**Table A17:** Brief description of the explained and explanatory variables, with their corresponding levels, considered in the experimental statistical design for early bacterial adhesion measurements, for each alloy and bacterial strain. (*) The contact time levels for *S. aureus* strain are 30, 60 and 180 min. But, for *E. coli* strain they are 30, 60 and 120 min.

Explained Variable (Response)	Explanatory Variables (Factors)	Levels (Code)
Bacterial Density	Contact time	30 min
		60 min 120/180 min *
	Treatment	Control
		Electrodeposited UV-irradiated Electrodeposited+UV-irradiated

To test for the existence of significant differences in the values of the bacterial density for each alloy and bacterial strain, a two-way analysis of variance (ANOVA) and subsequent Tukey’s multiple comparison were performed. The statistical *p*-values of the different analyses are shown in Table A18 and Table A19.

-------------------------------------------------------------------------

--- **ANOVA test for AZ31 alloy and *****S. aureus***** strain -----------**

> Anova (AnovaModel.1)

Anova Table (Type II tests)

Response: Density

              Sum Sq   Df  F value    Pr(>F)  

Contactime         32561    2   2250.66    < 2.2×10^−16^ ***

Treatment          7847    3   361.62    < 2.2×10^−16^ ***

Contactime:Treatment    6699    6   154.35    < 2.2×10^−16^ ***

Residuals          1476    204

---

Signif. codes: 0 ‘***’ 0.001 ‘**’ 0.01 ‘*’ 0.05 ‘.’ 0.1 ‘ ’ 1

--- **ANOVA test for MgCa alloy and *****S. aureus***** strain -----------**

> Anova(AnovaModel.2)

Anova Table (Type II tests)

Response: Density

              Sum Sq  Df    F value    Pr(>F)  

Contactime         85128   2    14.3848   0.0000014315 ***

Treatment         99171   3    11.1718   0.0000008075 ***

Contactime:Treatment    25360   6     1.4284    0.2052  

Residuals         603630   204            

---

Signif. codes: 0 ‘***’ 0.001 ‘**’ 0.01 ‘*’ 0.05 ‘.’ 0.1 ‘ ’ 1

--- **ANOVA test for AZ31 alloy and *****E. coli***** strain -----------**

> Anova(AnovaModel.3)

Anova Table (Type II tests)

Response: Density

             Sum Sq  Df    F value   Pr(>F)  

Contactime        56503   2    9218.79   < 2.2×10^−16^ ***

Treatment         4586    3    498.85   < 2.2×10^−16^ ***

Contactime:Treatment    2051   6    111.57   < 2.2×10^−16^ ***

Residuals         625    204           

---

Signif. codes: 0 ‘***’ 0.001 ‘**’ 0.01 ‘*’ 0.05 ‘.’ 0.1 ‘ ’ 1

--- **ANOVA test for MgCa alloy and *****E. coli***** strain -----------**

> Anova(AnovaModel.4)

Anova Table (Type II tests)

Response: Density

             Sum Sq   Df   F value    Pr(>F)  

Contactime        68213    2   4708.764   < 2.2×10^−16^ ***

Treatment         48111    3   2214.085   < 2.2×10^−16^ ***

Contactime:Treatment    2563    6   58.982    < 2.2×10^−16^ ***

Residuals         1478    204

---

Signif. codes: 0 ‘***’ 0.001 ‘**’ 0.01 ‘*’ 0.05 ‘.’ 0.1 ‘ ’ 1

**Table A18:** Summary of the *p*-values of the two-way ANOVA on bacterial density in the comparison between alloys and bacterial strains (at 95% confidence level).

*p* Values Two-Way ANOVA	Bacterial Density			
	AZ31/*S. Aureus*	MgCa/*S. Aureus*	AZ31/*E. coli*	MgCa/*E. coli*
Contact time	<0.0001 ***	0.0000014315 ***	<0.0001 ***	<0.0001 ***
Treatment	<0.0001 ***	0.0000008075 ***	<0.0001 ***	<0.0001 ***
Interaction	<0.0001 ***	0.2052	<0.0001 ***	<0.0001 ***

*** *p* ≤ 0.001.

**Table A19:** Summary of the statistical *p*-values for bacterial density and ANOVA residuals data after checking the normality and homocedasticity corresponding tests.

Data Studied	Normality *p*-Value (Kolmogorov-Smirnov Test)	Homocedasticity *p*-Value (Bartlett Test)
Bacterial Density	0.0859	0.1458
Residuals from ANOVA1	0.0421 *	0.1458
Residuals from ANOVA2	0.1147	0.1458
Residuals from ANOVA3	0.0965	0.1458
Residuals from ANOVA4	0.1587	0.1458

* *p* ≤ 0.05.
